# Hydrogeological typologies of the Indo-Gangetic basin alluvial aquifer, South Asia

**DOI:** 10.1007/s10040-017-1550-z

**Published:** 2017-02-23

**Authors:** H. C. Bonsor, A. M. MacDonald, K. M. Ahmed, W. G. Burgess, M. Basharat, R. C. Calow, A. Dixit, S. S. D. Foster, K. Gopal, D. J. Lapworth, M. Moench, A. Mukherjee, M. S. Rao, M. Shamsudduha, L. Smith, R. G. Taylor, J. Tucker, F. van Steenbergen, S. K. Yadav, A. Zahid

**Affiliations:** 1 British Geological Survey, Lyell Centre, Research Avenue South, Riccarton, Edinburgh, EH14 4AS UK; 2 Department of Geology, University of Dhaka, Dhaka, 1000 Bangladesh; 3 Department of Earth Sciences, University College London, Gower Street, London, WC1E 6BT UK; 4 International Waterlogging and Salinity Research Institute (IWASRI), Water and Power Development Authority, Lahore, Pakistan; 5 Overseas Development Institute, 203 Blackfriars Road, London, SE1 8NJ UK; 6 Institute for Social and Environmental Transition‐Nepal, Manasi Marga, Kathmandu Municipality‐4, Chandol, Kathmandu, Nepal; 7 Global Water Partnership, 25 Osberton Road, Summertown, Oxford, UK OX2 7NU UK; 8 National Institute of Hydrology, Roorkee, 247667 Uttarakhand India; 9 British Geological Survey, MacLean Building, Crowmarsh Gifford, Wallingford, Oxfordshire OX10 8BB UK; 10 Institute for Social and Environmental Transition‐International, 948 North Street 7, Boulder, Colorado 80304 USA; 11 Department of Geology and Geophysics, Indian Institute of Technology, Kharagpur, India; 12 Institute for Risk and Disaster Reduction, University College London, Gower Street, London, WC1E 6BT UK; 13 Filters for Families, 2844 Depew St., Wheat Ridge, Colorado 80214 USA; 14 Department of Geography, University College London, Gower Street, London, WC1E 6BT UK; 15 MetaMeta Research, Postelstraat 2, 5211 EA Hertogenbosch, The Netherlands; 16 Ground Water Hydrology, Bangladesh Water Development Board, 72 Green Road, Dhaka, Bangladesh

**Keywords:** Indo-Gangetic basin aquifer, Aquifer properties, Groundwater resource, Groundwater quality, Recharge

## Abstract

The Indo-Gangetic aquifer is one of the world’s most important transboundary water resources, and the most heavily exploited aquifer in the world. To better understand the aquifer system, typologies have been characterized for the aquifer, which integrate existing datasets across the Indo-Gangetic catchment basin at a transboundary scale for the first time, and provide an alternative conceptualization of this aquifer system. Traditionally considered and mapped as a single homogenous aquifer of comparable aquifer properties and groundwater resource at a transboundary scale, the typologies illuminate significant spatial differences in recharge, permeability, storage, and groundwater chemistry across the aquifer system at this transboundary scale. These changes are shown to be systematic, concurrent with large-scale changes in sedimentology of the Pleistocene and Holocene alluvial aquifer, climate, and recent irrigation practices. Seven typologies of the aquifer are presented, each having a distinct set of challenges and opportunities for groundwater development and a different resilience to abstraction and climate change. The seven typologies are: (1) the piedmont margin, (2) the Upper Indus and Upper-Mid Ganges, (3) the Lower Ganges and Mid Brahmaputra, (4) the fluvially influenced deltaic area of the Bengal Basin, (5) the Middle Indus and Upper Ganges, (6) the Lower Indus, and (7) the marine-influenced deltaic areas.

## Résumé

L’aquifère de l’Indus et du Gange est une des ressources en eau transfrontalière la plus importante au monde et un des aquifères le plus exploité au monde. Pour mieux comprendre le système aquifère, des typologies ont été caractérisées pour cet aquifère ; elles intègrent pour la première fois un jeu de données disponibles sur l’ensemble du bassin de l’Indus et du Gange à une échelle transfrontalière, et apportent une conceptualisation alternative de ce système aquifère. Traditionnellement considéré et cartographié comme un simple aquifère homogène aux propriétés aquifères similaires et comme une ressource d’eau souterraine à l’échelle transfrontalière, les typologies mettent en évidence des différences significatives spatiales de la recharge, de la perméabilité, de la capacité de stockage et de la chimie des eaux souterraines sur l’ensemble du système aquifère à une échelle transfrontalière. Ces changements sont systématiques coïncidant aux changements à large échelle de la sédimentologie de l’aquifère alluvial du Pléistocène et de l’Holocène, du climat et des pratiques récentes d’irrigation. Sept typologies de l’aquifère sont présentées, chacune ayant un ensemble distinct de défis et d’opportunités pour le développement des eaux souterraines et une résilience différente à l’exploitation et au changement climatique. Les sept typologies sont: (1) la marge de piedmont, (2) Le haut Indus et le Ganges moyen supérieur, (3) le Ganges inférieur et le Brahmapoutre moyen, (4) la zone deltaïque du bassin du Bengale sous influence fluviale, (5) l’Indus moyen et le Ganges supérieur, (6) l’Indus inférieur, et (7) la zone deltaïque sous influence marine.

## Resumen

El acuífero Indo-Gangético es uno de los recursos hídricos transfronterizos más importantes y el acuífero más explotado del mundo. Para comprender mejor el sistema acuífero, por primera vez se han caracterizado tipologías para el acuífero, integrando los conjuntos de datos existentes a través de la cuenca hidrográfica Indo-Ganges a una escala transfronteriza, y proporcionando una conceptualización alternativa de este sistema acuífero. Tradicionalmente consideradas y cartografiadas como un solo acuífero homogéneo de propiedades acuíferas y recursos de agua subterránea comparables a escala transfronteriza, las tipologías iluminan diferencias espaciales significativas en la recarga, permeabilidad, almacenamiento y química del agua subterránea a través del sistema acuífero a esta escala transfronteriza. Estos cambios son mostrados por ser sistemáticos, coincidentes con cambios en gran escala en la sedimentología del acuífero aluvial del Pleistoceno y del Holoceno, en el clima y en las prácticas recientes de riego. Se presentan siete tipologías del acuífero, cada una con un conjunto distinto de desafíos y oportunidades para el desarrollo del agua subterránea y una diferente resiliencia a la extracción y al cambio climático. Las siete tipologías son: (1) el margen del piedemonte, (2) el Indus superior y el Ganges superior-medio, (3) el Ganges inferior y el Brahmaputra medio, (4) el área deltaica fluvialmente influenciada de la cuenca de Bengala, (5) el Ganges superior, (6) el Indo Inferior, y (7) las áreas deltaicas con influencia marina.

## 摘要

印度-恒河含水层是世界上最重要的跨界水资源之一,也是世界上开采量最大的含水层。为了更好地了解含水层系统,结合现有的印度-恒河流域盆地数据,第一次在跨界尺度上对含水层的类型进行了特征描述,提供了这个含水层系统供选择的概念模型。传统上,被认为并被绘制为可比较含水层特性的单一均质含水层及跨界尺度上地下水资源,类型学阐明了这个跨界尺度上含水层系统补给、透水率、储存量和地下水化学上的重要空间差别。这些变化显示与更新世和全新世冲积含水层、气候和最近灌溉实践沉积学上的大尺度变化是系统性的、也是一致的。展示了含水层的七个类型,每个类型都具有一套独特的地下水开发的挑战和机会以及针对抽水和气候变化的不同恢复力。七个类型为:(1)山前边缘;(2)印度河上游及恒河中上游;(3)恒河下游及雅鲁藏布江中游;(4)孟加拉流域洪积影响的三角洲地区;(5)印度河中游及恒河上游;(6)印度河下游;(7)海相影响的三角洲地区。

## Resumo

O aquífero do Indo-Gangético é um dos recursos hídricos transfronteiriços mais importantes do mundo, e o mais explorado. Para melhor entender o sistema aquífero, tipologias foram caracterizadas para o aquífero, que integram pela primeira vez bancos de dados existentes sobre a bacia de abastecimento Indo-Gangética em uma escala transfornteiriça, e fornecem uma conceptualização alternativa desse sistema aquífero. Tradicionalmente considerado e mapeado como um aquífero homogêneo de propriedades aquíferas comparáveis e recursos subterrâneos em escala transfronteiriça, as tipologias elucidam diferenças espaciais significantes na recarga, permeabilidade, armazenamento, e química das águas subterrâneas pelo aquífero nessa escala transfronteiriça. Essas mudanças aparentam ser sistemáticas, concorrentes com mudanças em larga escala na sedimentologia do aquífero aluvial do Pleistoceno e Holoceno, clima e práticas recentes de irrigação. Sete tipologias do aquífero são apresentadas, cada uma tendo conjuntos de desafios e oportunidades distintos para o desenvolvimento das águas subterrâneas e uma resiliência diferente nas mudanças de extração e clima. As sete tipologias são: (1) a margem piemonte, (2) O Alto Indo e o Alto-médio Ganges, (3) O Baixo Ganges e o Médio Brahmaputra, (4) a área deltaica influenciada fluvialmente da Bacia de Bengala, (5) o Médio Indo e o Alto Ganges, (6) o Baixo Indo, e (7) as áreas deltaicas com influencia marinha.

## Introduction

**Fig. 1:**
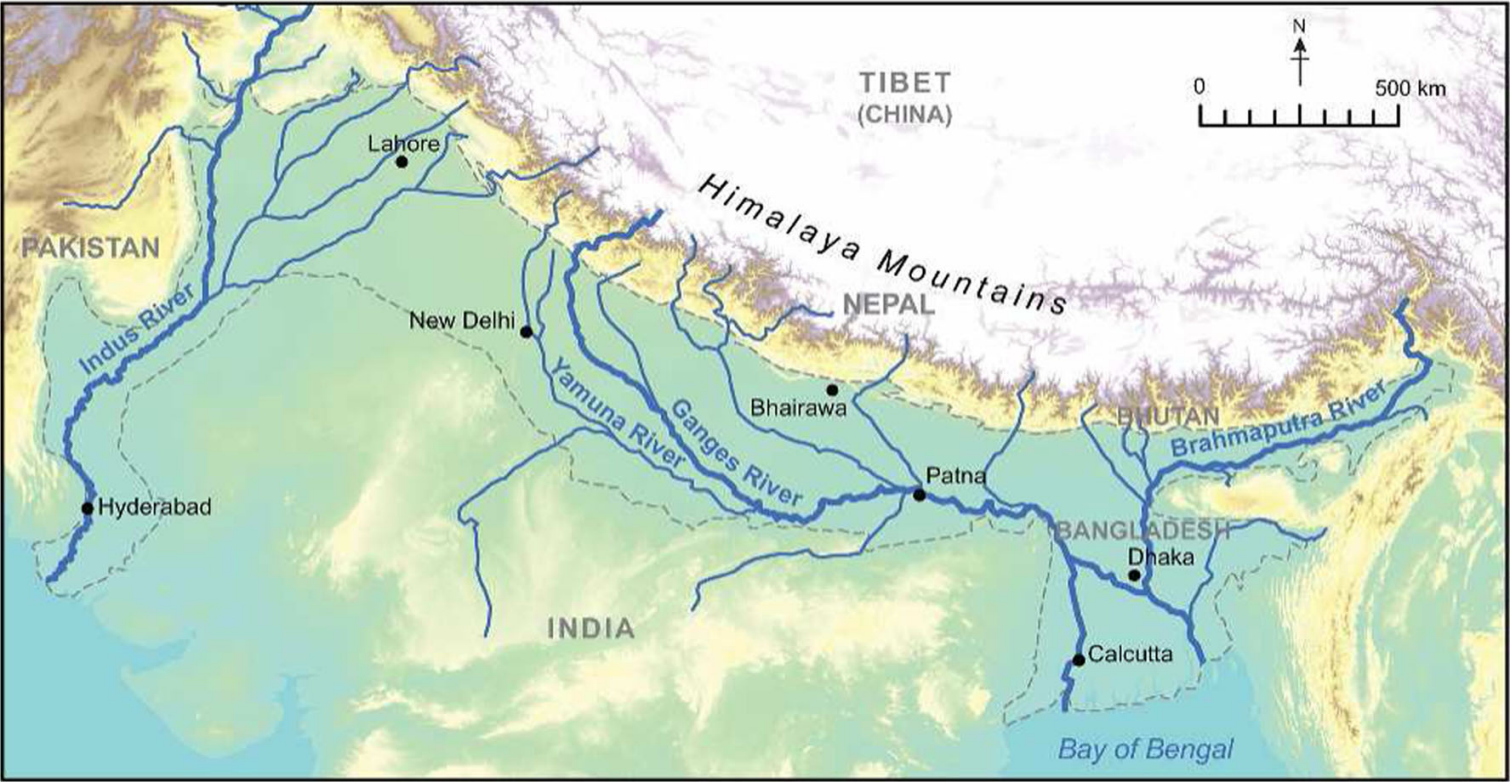
Location map of the IGB aquifer (topography as background)

The Indo Gangetic Basin alluvial aquifer system (known as the IGB aquifer throughout the rest of this report) is one of the world’s most important transboundary freshwater bodies (MacDonald et al. 2016). Groundwater abstracted from the alluvial aquifer system comprises approximately a quarter of the world’s total groundwater abstraction (Wada et al. 2010; Siebert et al. 2007) and underpins the agricultural productivity of south Asia (Shah 2009). Formed from sediments which have been eroded from the Himalayas and redistributed by the Indus, Ganges and Brahmaputra rivers, across the Indo-Gangetic plain in Pakistan, northern India, southern Nepal and Bangladesh, the aquifer system (Fig. 1) is characterized in most parts by low topographic relief, unconsolidated alluvium and shallow depth to groundwater. Abstraction is widespread and prolific due to groundwater accessibility and low cost of development. The aquifer is often represented on hydrogeological maps as a single highly permeable homogenous aquifer (Struckmeier and Richts 2008; CGWB 2012; Mukherjee et al. 2015); however, in practice the aquifer system is highly variable with significant spatial variability in groundwater recharge, permeability, storage and water chemistry.

Differences in aquifer conditions control the response of groundwater systems to the pressures of abstraction and climate change (Foster and MacDonald 2014). In the IGB aquifer of today, the many challenges include groundwater depletion (Rodell et al. 2009; Tiwari et al. 2009); salinization of shallow groundwater (Quereshi et al. 2008); mobilization of natural occurring arsenic (DPHE and BGS 2001); and increasing nitrate concentrations (CGWB 2010). To continue to develop and use groundwater, while minimising undesirable impacts, will require a clear understanding of the IGB aquifer system. Recognition and characterization of different typologies across the aquifer forms an important part of this process.

Here, typologies have been developed for the transboundary IGB aquifer by assembling and systemising the large amount of disparate existing data and information for the aquifer. The typologies provide a new lens through which to view the aquifer system and delineate areas with distinct sets of challenges and opportunities for groundwater development. The seven typologies are presented, alongside descriptions of the major characteristics of each, and the implications for groundwater governance. New basin-wide maps developed in the process of delineating the typologies are also presented: alluvium geology, aquifer permeability and storage, groundwater chemistry and recharge processes.

Typologies have been used in Europe to govern groundwater in accordance with the European Water Framework Directive (Vincent et al. 2002; Moss et al. 2003; Borja 2004). Typologies are used to delineate areas of aquifer systems, or groundwater ‘bodies’ which require specific management practices according to the aquifer properties (UKTAG 2011). It is of particular relevance in the case of the IGB aquifer that typologies have proved useful at a variety of scales, from continental (e.g. Wendland et al 2008) to national (e.g. Ó Dochartaigh BE et al. 2015) and regional (e.g. Garduño et al. 2009; Raj 2011). Transboundary typologies can provide apolitical evidence to inform transboundary aquifer management (Struckmeier et al. 2006).

## Methodology

**Fig. 2:**
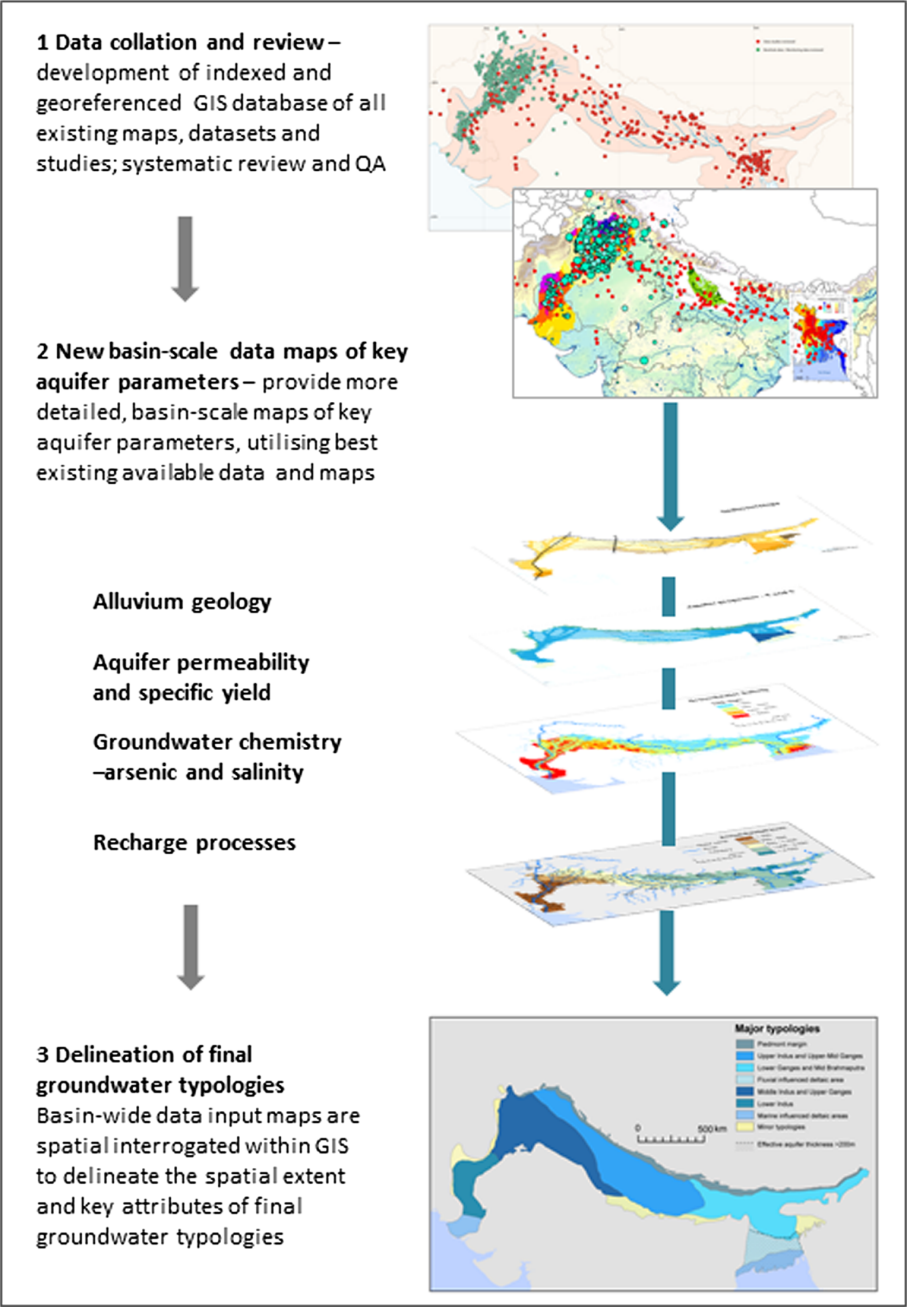
Framework and workflow through which the final typologies were developed. (*GIS* geographical information system; *QA* quality assurance)

The typologies were developed from the collation and integration of published studies and best-available national, regional and local-scale geological, sedimentological, hydrogeological, hydrochemical, hydrological and climatological datasets (Fig. 2). The work focused on the depths of the IGB aquifer in which groundwater exploitation is concentrated, which was taken to be the upper 200 m, except in the Bengal Basin where the aquifer is exploited at depths up to 350 m. Over 500 studies were reviewed in total: 56 of these focused on geological information; 415 hydrogeological studies; and 42 relating to climate and hydrological studies. These data were amalgamated by systematic review. Associated datasets of a comparable quality were integrated to develop new transboundary maps of key hydrogeological properties: aquifer permeability, storage, recharge, and groundwater chemistry. These maps were used to delineate the framework from which the final typologies were developed. The following section describes the systematic review process, the methods used to generate each of the basin-wide maps, and the final typologies in more detail.

### Data review process

Data and studies included within the review were identified by two methods. Publically available data from published and grey literature studies were identified from a series of Boolean Internet searches. Over two thirds of the 523 studies reviewed were sourced by this method, providing the majority of the qualitative data reviewed on the IGB aquifer geology, sedimentology and hydrogeology. Quantitative hydrogeological datasets, particularly of any substantial time series or spatial extent, are not readily accessible for the region, although many exist; key datasets were identified and collated in a collaborative effort between regional research partners. Whilst forming a minority of the total number of studies reviewed, these quantitative datasets were an essential component of the review, and the collation of these data enabled hydrogeological properties of the IGB aquifer to be parameterized at a transboundary scale and at a greater quantitative resolution than previously possible.

All 523 studies, including the quantitative datasets, were indexed, georeferenced and stored in a Microsoft Access database. ArcGIS (10.1) was used to spatially interrogate the different types of information held within the database. Data were systematically reviewed to apply a confidence rank to the studies and the data contained. Such a review process was essential to ensure that the combined hydrogeological datasets and maps developed were based on data generated by comparably robust methods, scale and data quality, despite being of multiple third-party provenances. Key criteria used in assigning a confidence value were: (1) that the depth and aquifer unit for the study was well constrained; (2) the date was available for temporally dependent data (e.g. water levels, groundwater chemistry); (3) sufficient methodological description was available to assess the quality of the data; and (4) the methods used to generate the data were appropriate. Data which only partially satisfied these criteria were ranked to be of lower confidence. High-quality studies were used as the key inputs to develop and parameterize the new basin-wide maps of each aquifer parameter, and lower quality studies were used only in areas where there were few or no high-quality studies to use. The review process identified 80 key benchmark studies which provided the highest quality systematic regional data and these formed the key datasets for the typologies.

### Development of new transboundary maps

#### Geological framework

Available regional and national geological maps, alongside the large number of published sedimentological studies, were used to develop a new geological map of the alluvium of the exploited IGB aquifer. This map provides higher-resolution information on the geological age and sedimentological characteristics of the alluvium of the effective aquifer across the IGB than previously available within transboundary geological maps of the aquifer system, which traditionally portray the aquifer alluvium as a single homogenous unit at this scale. Identifying this variability of the alluvium sedimentology at a transboundary scale provides a robust framework within which to consider changes in the hydrogeological properties of aquifer.

Existing available national and regional geology maps were used to develop the initial line work of the map, and were used to identify and demarcate the spatial extent of differing ages of alluvium across the aquifer. This line work was then validated and parameterized with information on modes of alluvium deposition, likely grain-size and sediment composition from the large amount of detailed information contained in the many high-quality georeferenced sedimentological studies across the IGB aquifer at local-scale. By combining these data, nine discrete units of alluvium characterized by key systematic changes in stratigraphy, were identified and mapped across the aquifer.

#### Variations in aquifer properties

Copious hydrogeological data are available at local-scales across the IGB aquifer. Over 400 hydrogeological studies and datasets were georeferenced and quantitative data used to identify spatial patterns in transmissivity, aquifer anisotropy and to develop a map of specific yield.

Spatial variations in transmissivity were mapped using a combination of primary data from pumping tests (mainly in Pakistan and India) and a review of local-scale hydrogeological studies and regional maps across the IGB aquifer. Values of transmissivity extracted from the studies were georeferenced and spatially interrogated in GIS. From this, broad areas of similar ranges in transmissivity were identified and delineated for both the Holocene and Pleistocene-aged alluvium. Due to the local-scale variability of the alluvium, areas of distinct transmissivity ranges were only delineated where transmissivity values varied consistently in comparison to an adjacent area by either >100% or <50%, and over a spatial area of ≥2,500 km^2^. Systematic patterns in aquifer anisotropy were identified from existing data and published studies within the Upper and Lower Indus basin (Bennett et al. 1969; Chilton 1986; Ahmed 1995), the main Ganges basin (Khan et al. 2014) and the Bengal basin (Michael and Voss 2009a, b).

A different approach had to be taken to map specific yield (Sy) across the basin due to the local availability of specific yield data. Grain-size distribution data available from sedimentological studies for the upper 200 m of alluvium (the effective aquifer) across the basin were used as a proxy for Sy and mapped across the basin. Values of specific yield were attributed to the different grain size ranges based on studies on specific yield for grain sizes within Bangladesh (BGS and DPHE 2001). This attribution and the spatial Sy distribution mapped was then validated against available published values of specific yield across the basin (Bennett et al. 1969; Sir Mott MacDonald and Partners Ltd. 1984; Chilton 1986; Ahmad 1993; CGWB 2010; Shamsudduha et al. 2011; Khan et al. 2014). Specific yield values, rather than storativity are mapped, in order to provide a quantitative estimate of the maximum potential volume of groundwater stored within the alluvium aquifer, assuming that any currently confined aquifers could potentially become unconfined and release more water. A similar approach was taken in calculating the volume of groundwater across Africa (MacDonald et al. 2012). Many of the boreholes currently accessing groundwater across the IGB experience unconfined conditions.

#### Groundwater chemistry

Salinity and arsenic are the two most significantly known water quality issues within the basin, and as such these were the foci of the review of groundwater chemistry (MacDonald et al. 2016). Groundwater salinity was mapped by compiling and georeferencing existing information from hydrogeological maps, and refining and validating this line of work with quantitative salinity estimates from specific local-scale data studies and literature. In Pakistan, the published hydrogeological maps and drainage atlas were used as a base map (WAPDA 2001; IWASRI 2005) and refined with specific information from additional studies and surveys. In India, the Central Ground Water Board (CGWB) survey of shallow groundwater quality (CGWB 2010) was used as the initial base map of saline groundwater within the effective aquifer, with further data from local studies used to validate and refine the final spatial demarcation. Within Bangladesh, a recent survey of specific electrical conductivity (Ravenscroft et al. 2009) and a national survey of water chemistry (BGS and DPHE 2001) were used as the main datasets to map areas of significant salinity variations. The suggested World Health Organization (WHO) and Food and Agriculture Organization of the UN (FAO) guidelines were used to map relevant categories of saline groundwater: the FAO classify water as non-saline where total dissolved solids (TDS) is <500 mg/L; slightly saline where TDS is 500–1,000 mg/L; saline where TDS is 1,000–2,500 mg/L; and moderately to highly saline where TDS is >2,500 mg/L (FAO 1992). The WHO suggest waters with TDS <1,000 mg/L are generally acceptable (WHO 2003).

Arsenic concentrations across the basin were mapped within India using available data and maps by state water resources agencies, the CGWB and available local datasets (e.g. Mahanta et al. 2012). Within Bangladesh the BGS and DPHE (2001) national hydrochemical survey data were augmented by large-scale studies by Ravenscroft (2007) and Amini et al. (2008). Due to the variable amounts of available data which exist across the basin, areas of elevated arsenic within groundwater (>100 μg/L) were mapped to be ‘known’ where identified in detailed regional studies, or ‘likely’ where indicated in quantitative data available from georeferenced local studies.

Information on other water quality issues, such as naturally occurring elevated concentrations of fluoride, iron, manganese and uranium, and anthropogenic pollution from urban and agricultural activities, are not available over a sufficient extent of the aquifer for basin-wide maps to be developed, but these issues can be locally important. The typologies do however provide a basis for predicting where these other water quality issues may occur, based on the information assimilated in the data review.

#### Recharge

Recharge to the IGB aquifer occurs through several main mechanisms: rain-fed recharge, leakage from rivers, and leakage from canals. Other mechanisms can also be important in some areas, such as direct irrigation returns, and recharge induced by groundwater abstraction. A map was developed to show where each of the different recharge mechanisms could occur, but no attempt was made to estimate the average annual recharge across the basin collectively from these processes, or the significance of the different recharge processes within any one area. Rainfall for the basin was taken from the Climatic Research Unit (CRU) datasets for the years 1950 to 2012 (Jones and Harris 2013) and maps of average annual rainfall and number of rainy days were developed. The extent of rivers and canal networks were mapped within GIS using a variety of different georeferenced regional map datasets sourced by in-country partners, and validated on Google® Earth. In areas of high rainfall (>1,000 mm) recharge can be dominated by rainfall. In areas of low rainfall, (<500 mm) the recharge will be dominated by irrigation returns and canal leakage. In areas of moderate rainfall (500–1,000 mm) recharge is likely to be met by a combination of canal leakage and rainfall recharge.

### Developing the typologies

Seven main typologies were developed on the basis of key differences in hydrogeological properties across the aquifer system. Four accompanying minor typologies are delineated at the margin of the basin.

The typologies were demarcated by combining the maps of alluvium geology, permeability, storage, water chemistry and recharge processes within a GIS. Three distinct characteristics were used to delineate the final typologies: physical aquifer properties (based on the geological framework); groundwater chemistry (arsenic and salinity); and the dominant recharge process. Areas with similar properties were digitized to illustrate the extent of each typology.

The report provides a résumé of the new basin-wide understanding of the IGB aquifer developed from the review, new transboundary maps developed of the key properties and the deduced typologies. The major characteristics of each the typology are described alongside the implications for groundwater governance.

## New basin-wide datasets of the IGB groundwater system

### Geology of the alluvial aquifer

The Indo-Gangetic basin developed 15 million years ago in response to uplift of the Himalayan plateau with lithospheric loading and depression of the Indian continental plate (France-Lanord 1993; Kumar et al. 2014). The present day basin is an asymmetric trough, hosting vast sediment thicknesses (Singh 1996; Srivastava et al 2015) and the basin remains the world’s largest area of modern alluvial sedimentation (Sinha et al. 2014).

**Fig. 3:**
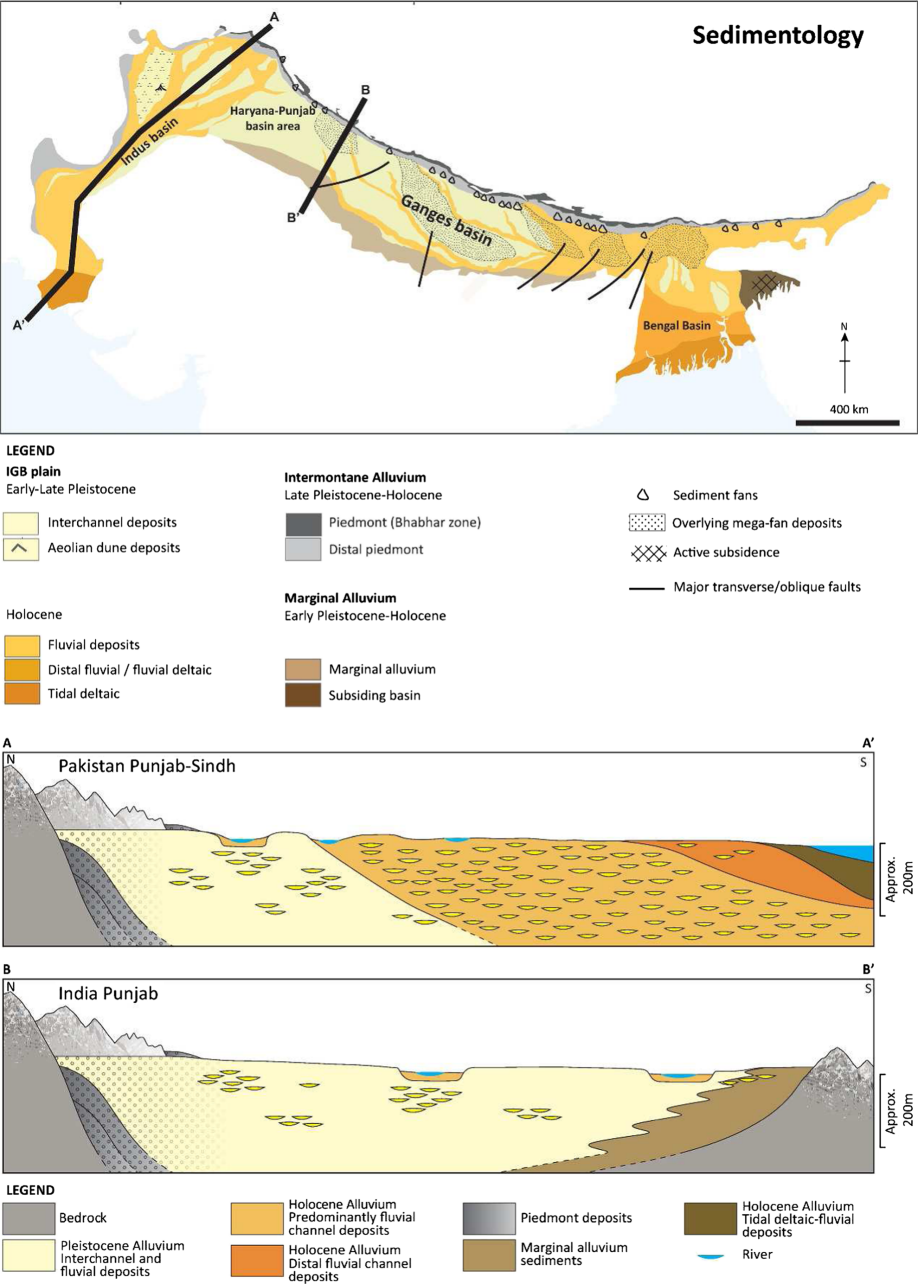
Sedimentology of the effective IGB alluvial aquifer. Cross-sections A–A′ and B–B′ illustrate the systematic variations in the alluvium stratigraphy within the effective aquifer within the Indus and upper Ganges basin, respectively. (Coastline outline provided by ESRI)

The basin is a foredeep depression, which has formed in front of the Himalaya as a result of the orogenic uplift and loading, and contains two main basin systems: the Indus basin in the west which deepens longitudinally away from the Himalaya towards the coast; and the Ganges basin in the central and eastern part of the foredeep which deepens transverse to the Himalaya towards the Bay of Bengal (Valdiya 2002; Singh 2007; Fig. 3). The intervening Haryana-Punjab basin area represents a shallower, over-filled region of the foredeep (Singh 1996). Collectively these three basin areas form the IGB. Continued convergence of the Indian plate at a rate of 2–5 cm/year, has driven uplift of the basin floor in fault-bounded blocks, generating basement highs in several areas of the Ganges basin where basin depth is now less than 1 km and it has affected the position of modern river courses (Singh 2007; Sahu and Saha 2014).

Sediment deposition has occurred since Late Cretaceous times with uplift and erosion of the Himalaya (Johnson and Alam 1991; Singh 1996; Alam et al. 2003). The bulk of the sedimentation which is relevant to the present-day aquifer has been deposited over the last 2 million years during the Pleistocene and Holocene (Singh 1996; Fig. 3). Groundwater exploitation is generally focused to the upper 200 m of these sediments across much of the basin, except in the Bengal Basin where the alluvial aquifer is exploited at depths up to 350 m.

### Sedimentology

At local scales the alluvial aquifer is highly complex, composed of alternating coarse and fine sands, silts and occasionally clays, deposited within sequences of channel and inter-channel deposits. These sediments form laterally discontinuous packages, each rarely more than a few kilometres across, and any one individual unit is generally less than 50 m thick (Sinha et al. 2005a; Samadder et al. 2011). At larger scales, distinct systematic changes in the sedimentology exist which are significant to the hydrogeology of the IGB aquifer. These differences in alluvium sedimentology have been largely driven by the proximal-distal changes in fluvial processes which have operated across the basin over time. Distinguishing between the Pleistocene and Holocene-aged alluvium, and where they form the effective alluvium aquifer within different parts of the IGB aquifer, provides a useful means of identifying the most significant of these changes and differences within the effective alluvium aquifer. The most important sedimentological variations within the IGB aquifer properties are: alluvium grain size; the ratio of channel to inter-channel deposits within the stratigraphy; and, the spatial and vertical distribution of the Pleistocene versus Holocene alluvium across the basin.

#### Alluvium grain size:

Alluvium grain-size changes from generally coarse gravel and sands (85% sands and gravels), up to boulder-sized, close to the mountain margins of the IGB aquifer and within megafan deposits (Day 1971; Shukla et al. 2001; Singh 1996; Singhal et al. 2010), to medium sands (70% fine-medium sands) within the central parts of the Ganges and Indus basin plains (Singh et al. 1999; Saha et al. 2011), to finally silt dominated (70% silts) deltaic deposits in the coastal regions (Goodbred and Kuehl 2000; BGS and DPHE 2001; Acharyya 2005; Fig. 3). This distinct downstream fining of alluvium sediment grain size from proximal to distal parts of the IGB basin reflects the progressive reduction in energy of river systems depositing the Pleistocene and Holocene alluvium sediment with increasing distance from the Himalaya.

#### Ratio of channel to inter-channel deposits:

The ratio of channel to inter-channel deposits within the Pleistocene and Holocene alluvium also changes across the IGB aquifer from proximal to distal areas, and is highly significant to the proportion of higher permeability sand deposits, to lower permeability silts and muds, which comprise the aquifer in different regions, and the anisotropy of the alluvial aquifer system.

Within the upper and central parts of the IGB basin, rivers systems are incising and of lower lateral aggradation than further downstream (Singh 1996). As a result, coarser, higher permeability channel deposits in the effective aquifer within this part of the IGB are spatially clustered, both vertically and laterally, within the stratigraphy, and there are significant areas and sediment thicknesses of lower permeability inter-channel deposits which contain a higher proportion of finer sands and silts (Sinha et al. 2005a; Clift and Giosan 2013). Downstream, within the central and lower parts of the IGB, fluvial systems show greater lateral aggradation and braiding as a result of the reduced hydraulic gradients to the coast (Sinha et al. 2005). Within these central and lower parts of the IGB the effective aquifer is composed of a larger proportion of channel deposits but also exhibits an increasingly prevalent inter-layering of lower permeability finer sands and silt deposits as a result of the lower energy of the fluvial processes. Within the coastal regions of the IGB, these trends continue and the effective aquifer is of much higher anisotropy. Here the alluvial sediments are predominantly composed of very fine sands and silts as a result of the lower energy of the fluvial system, and the sediments are highly stratified with low permeability silts and clays interbedded with fine sands on a 1–10s of metres scale.

#### Distribution of Pleistocene and Holocene alluvium:

The Pleistocene and Holocene alluvium have a distinct distribution within the IGB aquifer, which underpins many of the systematic changes in aquifer properties from proximal to distal areas in the aquifer system. In the upper and central parts of the IGB the effective aquifer is predominantly composed of Pleistocene-aged alluvium, as result of the strongly incising nature of rivers. Recent-Holocene aged alluvium is only deposited within narrow terraces (km wide) adjacent to active rivers (Sinha et al. 2005a, b; Clift and Giosan 2013; Fig. 3). The Pleistocene sediment is also, therefore, comprised predominantly of inter-channel deposits (sand and silt dominated), with clustered (laterally and vertically) coarse channel deposits due to the lower lateral aggradation of the fluvial systems in this part of the basin (Singh et al. 1999, 2005a). The sediment contains mainly oxidized coarse sands and silt components (Saha et al. 2011). Within the central and lower parts of the IGB the river systems change to become of much greater lateral aggradation, with numerous sinuous channels active at any one time. Here a significant thickness (>100 m) of Holocene sediment overlies Pleistocene alluvium (Sinha et al. 2005b) so that the Holocene alluvium forms the effective aquifer unit (Fig. 3). As a result of the different fluvial geomorphology, the Holocene alluvium is comprised predominantly of channel (medium sand-dominated to silt) deposits and the alluvium sediment is generally unoxidized (Singh 1996). These sedimentological differences between the Pleistocene and Holocene alluvium, and their distribution within the aquifer, alongside proximal-distal changes in sediment size, and changes in recharge processes and groundwater chemistry underpin the large-scale systematic changes in hydrogeology of the IGB aquifer and the groundwater typologies.

### Alluvium aquifer properties

The large-scale changes in alluvium sedimentology give rise to predictable and significant changes to the permeability, storage and anisotropy of the IGB aquifer at a basin-scale.

#### Permeability

**Fig. 4:**
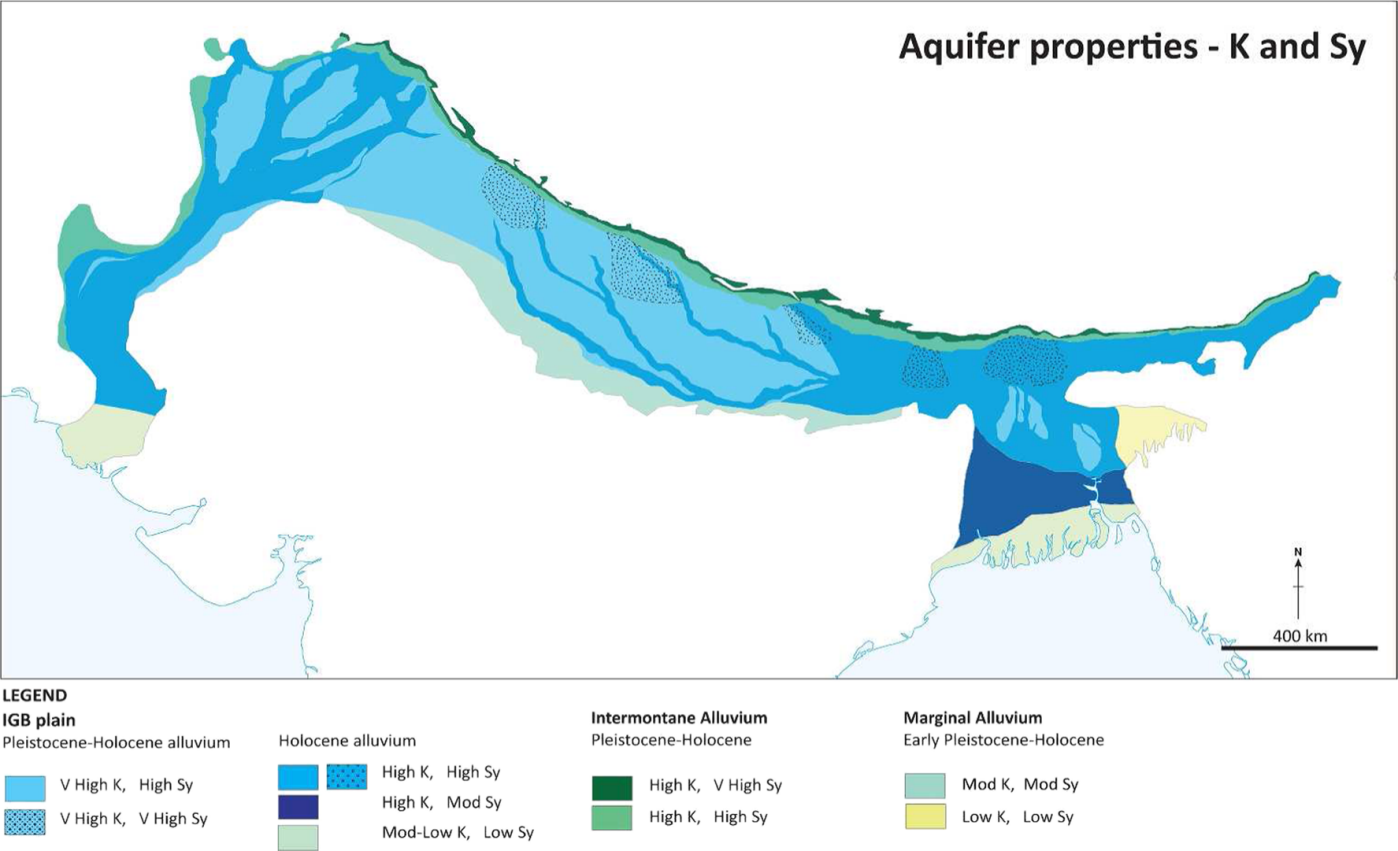
Aquifer properties of the IGB aquifer, which vary systematically on a basin-scale with the sedimentological characteristics of the Plio-Pleistocene–Holocene alluvium. (Coastline outline provided by ESRI)

**Fig. 5:**
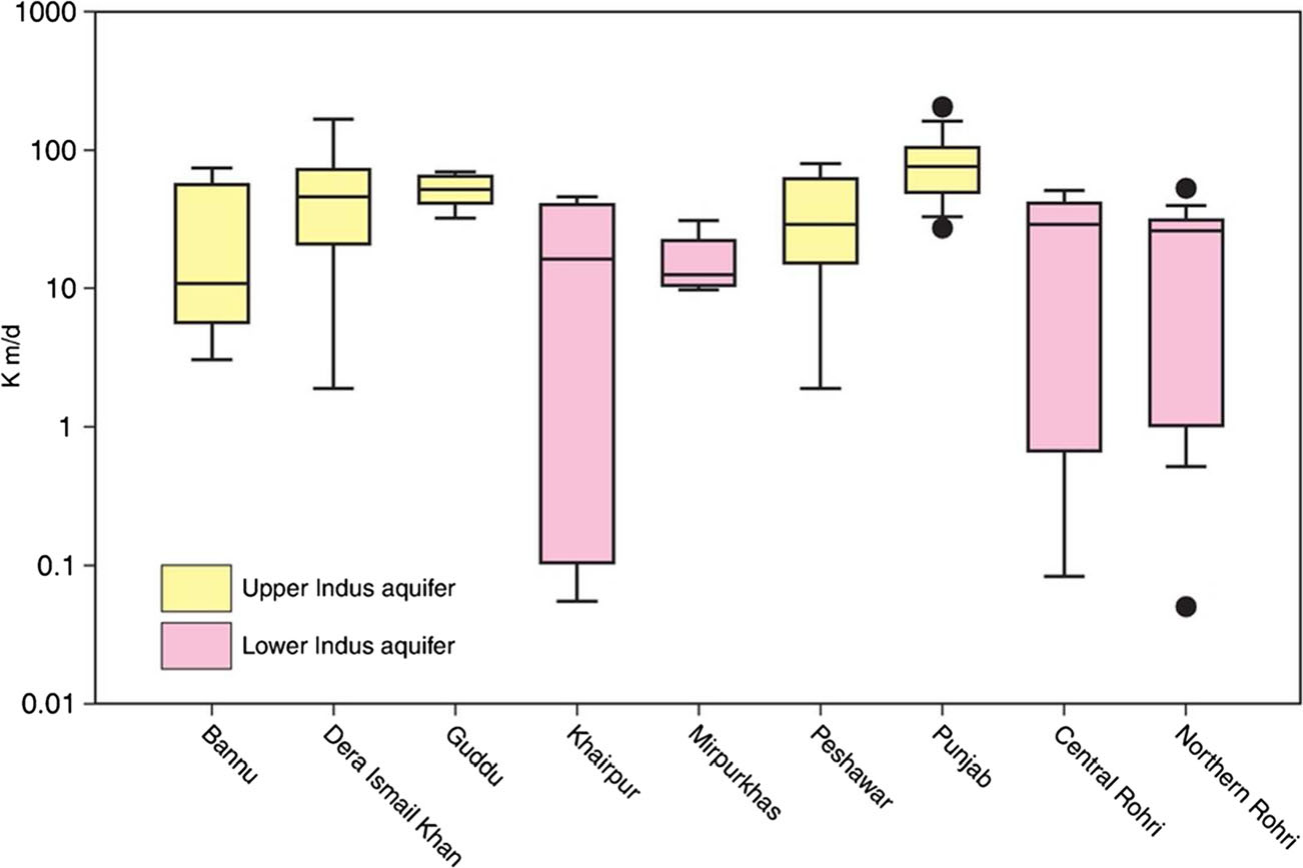
Estimated permeability (*K*) from pumping tests in the Indus

A marked proximal to distal reduction in permeability in the basin aquifer system is clearly shown from pumping-test data within both the Indus and Ganges basins in Bangladesh, concurrent with fining of the alluvium sediment (Figs. 4 and 5). In the Indus, hydraulic conductivity decreases from >60 to < 10 m/d from the upper to lower basin. These data derive from pumping tests carried out in tube wells generally <100 m deep and data from previous studies by Bennett et al. (1969), Ahmad (1993), Khan et al. (2008) and additional data from WAPDA. In the Ganges basin, a similar systematic proximal to distal trend in permeability is shown from pumping-test data within Bangladesh with hydraulic conductivity reducing from >50 m/d close to the Himalaya to less than 20 m/d near to the coast (Shamsudduha et al. 2011).

Permeability variation within the alluvial aquifer in the upper and central Ganges, however, is more complex, with inputs of coarser sediment occurring along most of the length of the central part of the Ganges alluvial aquifer from the Himalayan front (Fig. 4). Transmissivity determined from pumping tests in successful tube wells from Uttar Pradesh to Bihar in this part of the aquifer vary from several 100 to >5,000 m^2^/d, with median values around 3,000 m^2^/d, and with little evidence of a consistent downstream trend. Such transmissivity values correspond to hydraulic conductivity values of 5–100 m/d reported by the CGWB (2010). A similar range in permeability values is also derived from pumping tests from the Brahmaputra alluvial aquifer system which runs transverse to the Himalayan mountain front further east within Assam.

#### Specific yield

**Fig. 6:**
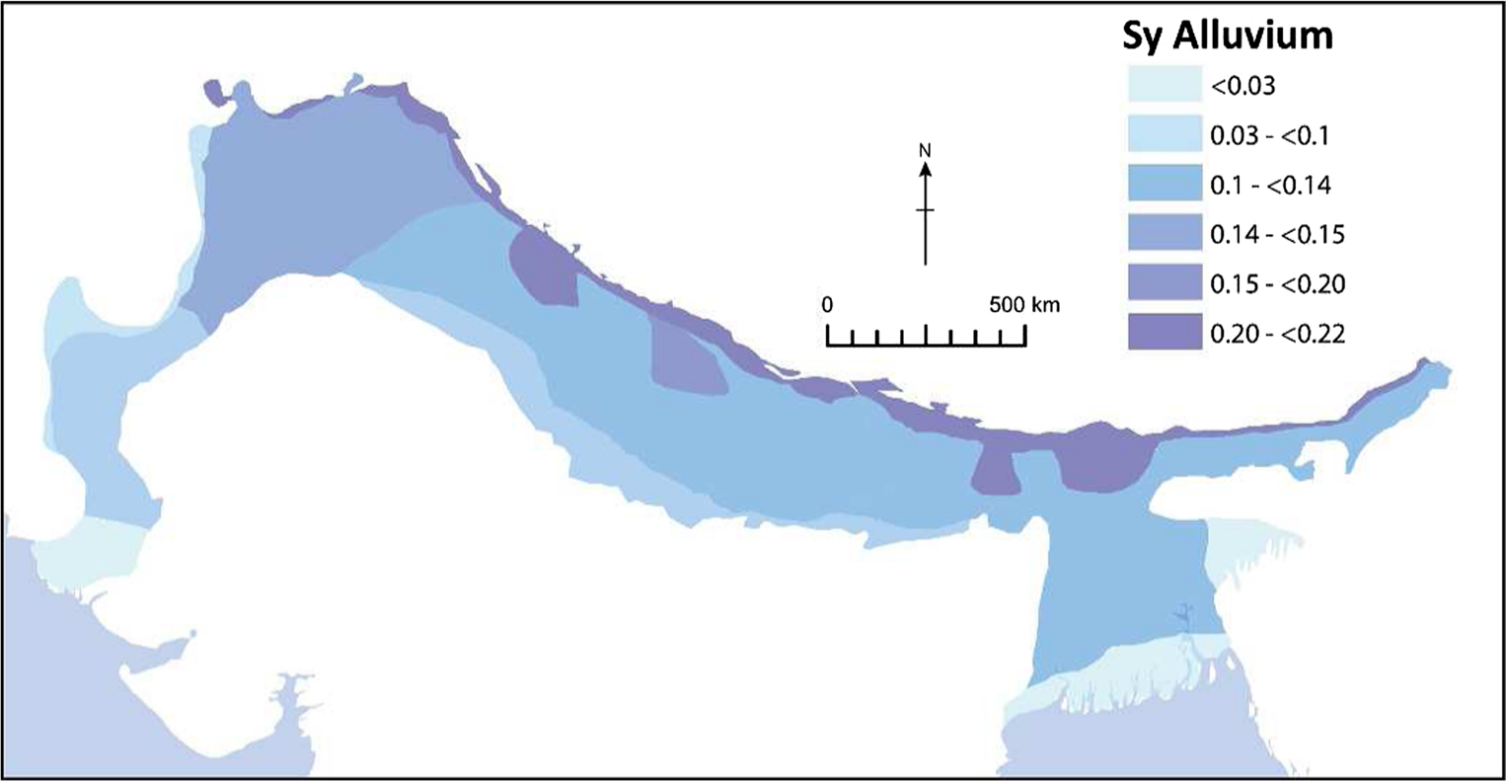
Variations in average specific yield (*Sy*) across the IGB (figure modified from MacDonald et al. 2016). (Coastline outline provided by ESRI)

The general variation in specific yield for the top 200 m of the IGB aquifer is shown in Fig. 6. Specific yield declines by an order of magnitude from proximal to distal areas in the IGB alluvial aquifer system. Specific yield is highest in the piedmont and large megafans where grain sizes and porosity are high, although overall aquifer thickness here is often less than elsewhere in the basin. For much of the basin specific yield is in the range 0.1–0.15. In the delta areas, specific yield is <0.05 due to the increase in silt content. Greatest potential volumes of groundwater stored within the alluvium aquifer are therefore indicated to be within the upper, middle and lower Indus and Ganges (Fig. 10), beyond the mountain margins.

#### Heterogeneity and anisotropy

Anisotropy in aquifer properties is an important control to the ratio of horizontal to vertical groundwater flow within aquifers. Following the trends in aquifer permeability and specific yield, the anisotropy of the IGB aquifer increases from proximal to distal areas, as a result of greater vertical heterogeneity of the alluvium sediment, and increased proportion of low permeability horizons on a 10s-of-metres scale within the more distal aquifer basin areas.

Studies in the Upper Indus suggest a ratio of bulk horizontal to vertical permeability of approximately 25 (Bennett et al. 1969), rising to 50–100 in the main Ganges basin (Khan et al. 2014), and 200–500 in the Lower Indus (Chilton 1986; Ahmed 1995). Away from these main basin areas, the anisotropy ratio is much higher: estimated to be 10,000 in the southern Bengal Basin, and 20,000 in the coastal areas (Michael and Voss 2009a, b). Limited data from modern sediments close to the major rivers show a much lower anisotropy ratio of <10 (Ahmed 1995).

The anisotropy ratios across the main basin areas away from the coastal areas are supported by the high rate of drilling success across much of the alluvial aquifer system, with sufficient thickness of sand usually encountered in a 100-m-deep tubewell to provide a satisfactory yield. For example, within the main Ganges basin and the Upper Indus, lower permeability layers are common and encountered in most tubewells, but since they are laterally discontinuous, groundwater can still move deep within the sequence in response to vertical hydraulic gradients induced by pumping (Lapworth et al. 2015). Bulk permeability and specific yield are high in these areas, and pumping rates of 10–100 L/s are easily sustained. Closer to the coast, finer-grained sediments become predominant and more continuous so vertical hydraulic continuity is more restricted.

### Groundwater chemistry: salinity and arsenic

Two of the greatest constraints to the useable volume of groundwater in the IGB are the presence of saline water at shallow depths and elevated arsenic concentrations. Information on other water quality issues which can be important at local scales, such as naturally occurring elevated concentrations of fluoride, iron, manganese and uranium, and anthropogenic pollution from urban and agricultural activities, is not available over a sufficient extent of the aquifer and therefore is not discussed further here.

#### Groundwater salinity

**Fig. 7:**
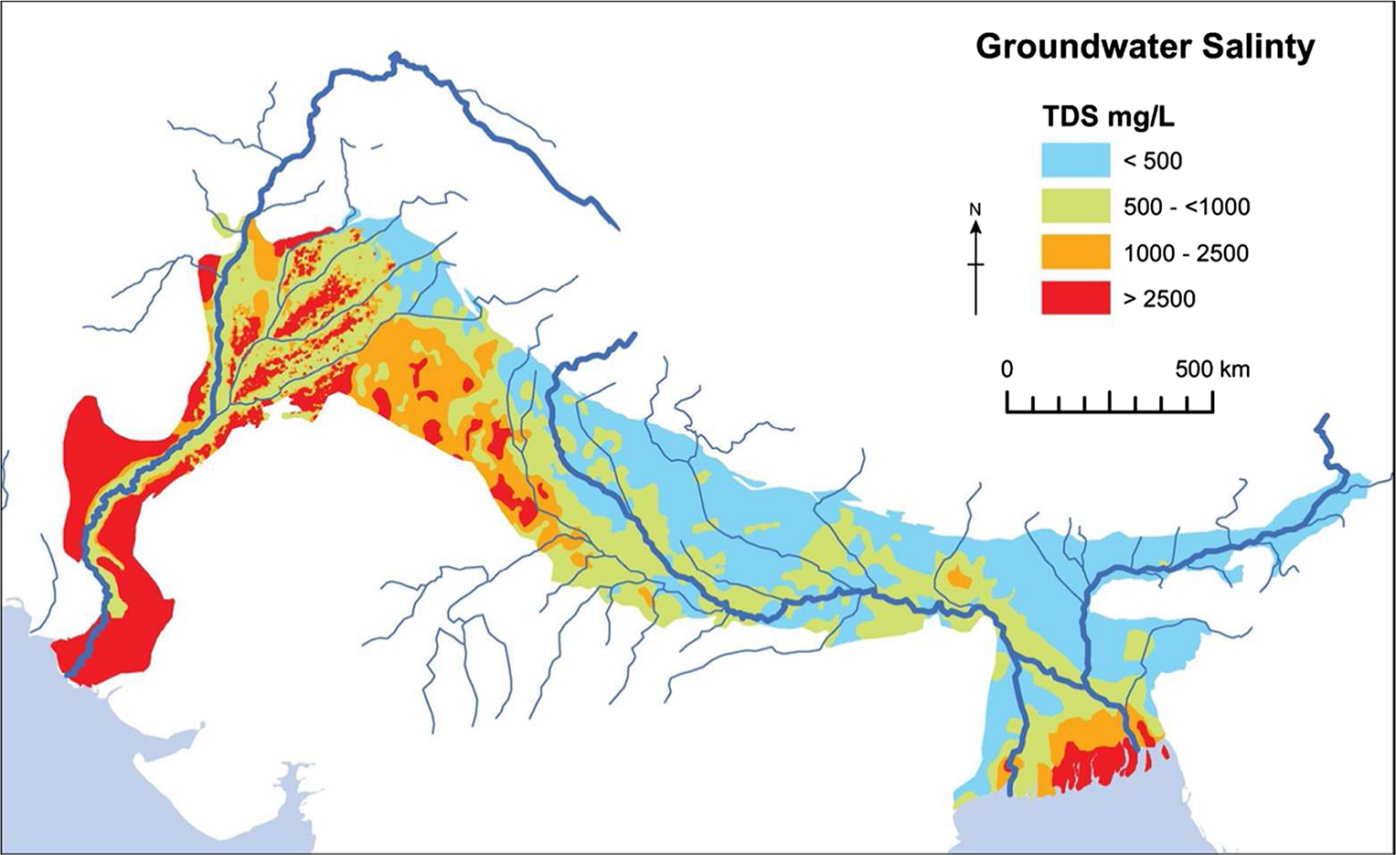
Distribution of groundwater salinity in the top 200 m of the IGB aquifer (figure modified from MacDonald et al. 2016). (Coastline outline provided by ESRI)

The distribution of saline water in the top 200 m of the IGB aquifer is shown in Fig. 7. Saline groundwater at over 1,000 mg/L total dissolved solids (TDS) is present across approximately 28% of the aquifer area; the most-affected areas are located along the coastal margins and within the Indus and Upper Ganges basin areas of the aquifer system. For drinking water, the WHO suggests waters should have less than 1,000 mg/L TDS to be acceptable to consumers (WHO 2003). For agriculture uses, there are no strict definitions for the use of saline water: the FAO classify water as non-saline at less than 500 mg/L, slightly saline from 500 to 1,500 mg/L and moderately saline from 1,500 to 7,000 mg/L (FAO 1992). Different crops have different tolerances to salt and the use of water beyond 1,000 mg/L must be carefully managed to sustainably farm without damaging the soil.

The origin of the salinity is complex (Schroder 1993; Goodbred 2003; Quereshi et al. 2008). It can arise from a combination of natural processes and is exacerbated in some areas by centuries of irrigation. The IGB basin has not been subject to widespread marine transgression (Schroder 1993; Valdiya 2002; Goodbred 2003) and the salinity in the Indus basin and Upper Ganges is almost entirely due to terrestrial processes. Only in the coastal regions of the Bengal Basin and Pakistan is there evidence of historical and current marine influence (Schroder 1993; BGS and DPHE 2001).

Across much of the basin, away from the coastal areas, saline groundwater is a consequence of high evaporation relative to rainfall. Current shallow water tables, irrigation or flooding can lead to high evaporation and consequent salinization of soil and groundwater. Pumping can also mobilize water from deeper in the sequence which can be saline due to the presence of evaporite sequences and rock–water interaction with the longer residence times of groundwater at depth. The distribution of evaporite deposits within the aquifer is largely governed by historical climate, with extended dry periods, or a succession of wet and dry periods, leading to their development (Valdiya 2002). The overall relative distribution of rainfall in the basin is thought to have remained relatively consistent (Goodbred 2003; Clift and Giosan 2013), with higher rainfall occurring closer to the Himalayas coupled with a west to east trend of increasing rainfall, there is therefore a greater likelihood of encountering evaporite sequences at depth in the currently drier areas of the basin, as evidenced by the presence of kankar deposits.

Modern irrigation practices have accentuated the natural patterns of saline groundwater in the IGB. Most of the Indus and upper and central parts of the Ganges basin aquifer are covered in a dense canal distribution network for agriculture irrigation. High rates of irrigation losses from these canals can lead to the development of small freshwater lenses in areas with generally saline groundwater; however, where the leakage and irrigation leads to very shallow water tables (such as in the Lower Indus), waterlogging and increased salinization can occur. Over-irrigation can also lead to degradation of groundwater quality (e.g. Ó Dochartaigh et al. 2010) where salts accumulated in the soil from evaporation are flushed through into the groundwater system.

Rivers also modify the distribution of the saline groundwater in the IGB aquifer. Rivers can flush out saline water to provide fresh groundwater close to the rivers. This dynamic is most apparent along the length of the River Indus and its tributaries where a buffer zone of 50 km alongside the rivers tends to have low saline groundwater (Fig. 7).

#### Arsenic

**Fig. 8:**
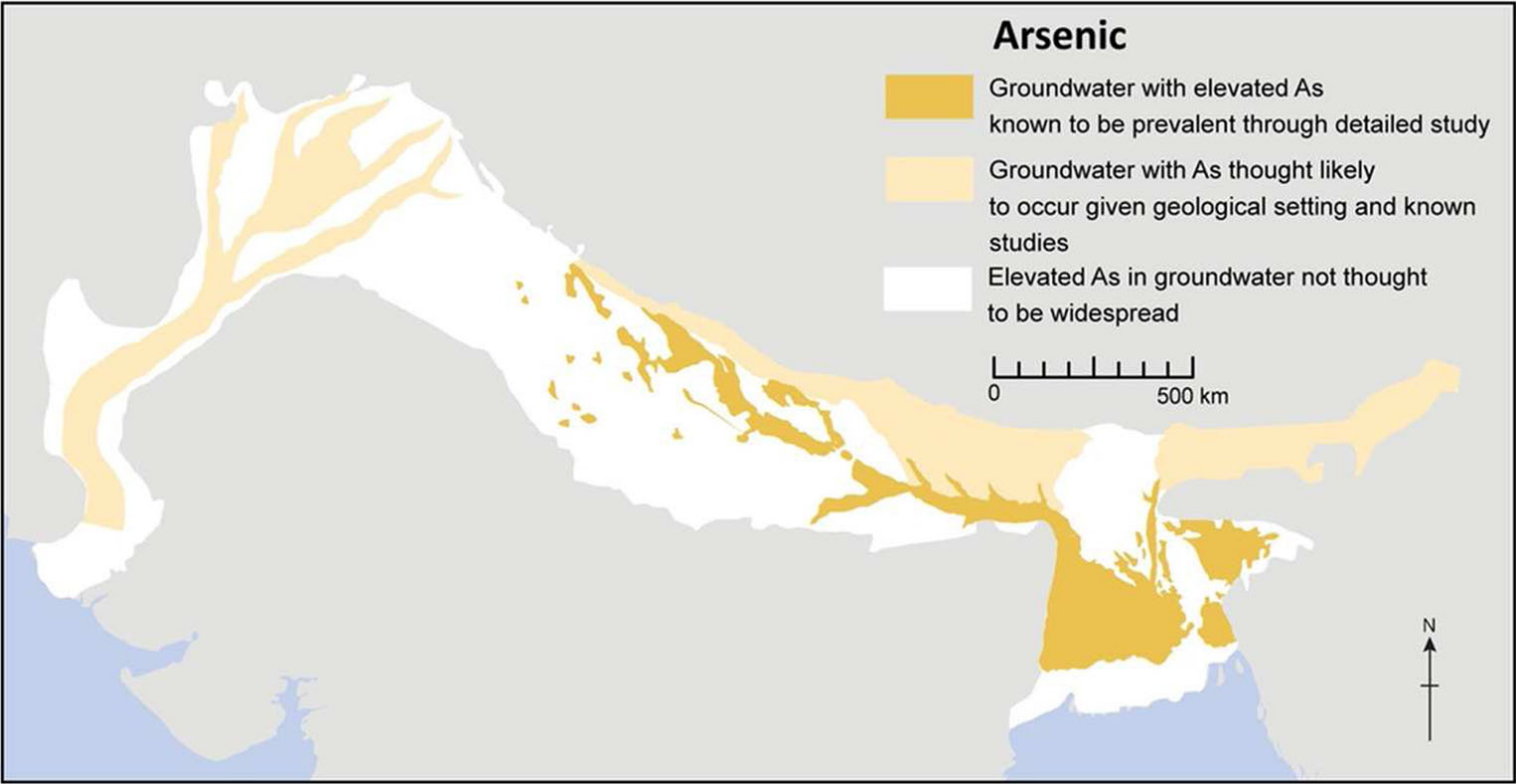
Known areas with elevated arsenic in the IGB aquifer system. Very high arsenic concentrations (>100 μg/L) are mostly restricted to groundwater in the uppermost 100-m alluvium in the southern Bengal Basin. Less extreme arsenic concentrations, though still >10 μg/L, are known to occur in other areas of the aquifer, including: Assam, southern Nepal, the Sylhet trough in eastern Bangladesh, and within Holocene sediments along the course of the Ganges and Indus river systems (figure modified from MacDonald et al. 2016). (Coastline outline provided by ESRI)

The distribution of elevated arsenic within the IGB aquifer is shown in Fig. 8. High concentrations of naturally occurring arsenic are most prevalent and widespread in the Bengal Basin, where it is commonly present at >100 μg/L and it places a major constraint on the development of groundwater. The arsenic-rich groundwater occurs in the chemically reducing, grey-coloured, Holocene sediments in the uppermost 100 m of the effective aquifer across the floodplains in the southern Bengal Basin (Fig. 8). Arsenic is thought to result from a higher proportion of argillaceous material and organic matter associated with the fine-medium sands of the Holocene alluvium, which produces reducing groundwater conditions, and within which, historic flushing of groundwater has been limited (McArthur et al. 2004; Shamsudduha et al. 2015). Many millions of people have developed symptoms of arsenic poisoning from use of the shallow groundwater for drinking water supply since the 1980s in this region (Ravenscroft et al. 2004). Approximately half the shallow hand-pumped wells in Bangladesh provide groundwater with 10–1,000 μg/L As (BGS and DPHE 2001 2001; BAMWSP 2004; BBS/UNICEF 2011).

Less extreme arsenic concentrations, though still >10 μg/L, occur in other parts of the IGB aquifer including Assam (India), southern Nepal, the Sylhet trough in eastern Bangladesh, and within Holocene sediments along the course of the Ganges in northern India, and also within the Indus system (Fig. 8). Throughout the IGB aquifer, arsenic concentrations are generally <10 μg/L within the Pleistocene alluvium sediments. Groundwater deeper than 150 m within these sediments has become a popular target of development in response to the arsenic crisis in the Bengal Basin. High-yielding deep wells have been installed in many rural water supply schemes and provincial towns in southern Bangladesh and West Bengal, India. Indeed these are the only areas in the IGB where the deeper groundwater resource is widely exploited. Recent investigations have raised concern for the security of deep groundwater pumping against invasion by arsenic drawn down from shallow levels (Michael and Voss 2008). Modelling studies have highlighted the need for more measurements of groundwater head in the deep regions of the Bengal Aquifer System (Ravenscroft et al. 2004; Michael and Voss 2009b; Burgess et al. 2010) and further research is needed to ascertain the hydraulic connection between the shallow and deeper groundwater resource in these areas.

### Recharge

**Fig. 9:**
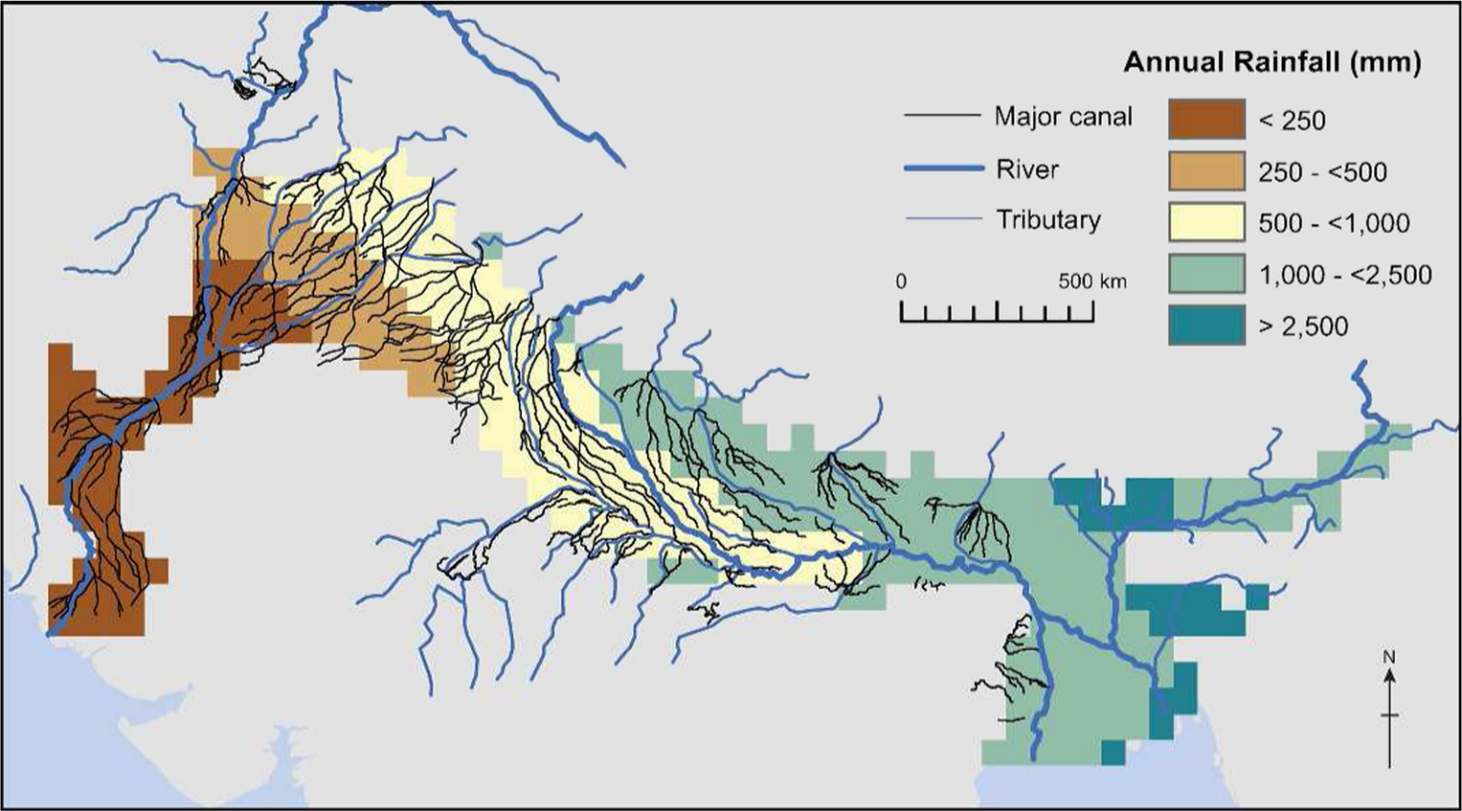
The distribution of factors affecting recharge processes across the IGB aquifer, including annual rainfall, river flows and irrigation canals (figure modified from MacDonald et al. 2016). (Coastline outline provided by ESRI)

Recharge to the IGB aquifer is complex and often controlled by several factors in any one place including rainfall volumes and intensity, canal leakage, irrigation returns, river inundation and even abstraction. Three datasets relating to the main recharge processes of rainfall, rivers and canal leakage were developed to understand how groundwater recharge may systematically vary across the basin. Figure 9 illustrates the highly complex pattern of recharge processes across the IGB aquifer which results from the combination of these three key factors.

#### Rain-fed recharge

There is considerable evidence of high rates of groundwater recharge from seasonal rainfall within the aquifer. Where average annual rainfall is greater than 1,000 mm, such as along the northern, central, and eastern parts of the IGB basin, direct, rain-fed recharge generally dominates (Lapworth et al. 2015). Rain-fed recharge can, however, occur even where annual rainfall is as low as 250–500 mm due to the intensity of individual rainfall events and permeability of soils. Studies of groundwater-level variations and rainfall in India led to an empirical formula being developed relating rainfall to recharge (Chaturvedi 1973) which has been modified by others (e.g. Kumar and Seethapathi 2002) and tested using environmental tracers (e.g. Goel et al. 1977; Datta and Goel 1977). Groundwater recharge is found by these studies to be negligible in areas with average annual rainfall below approximately 350 mm, less than 10% for 350–500 mm and then increases to between 10 and 20% of rainfall above 500 mm. These local studies give significantly higher values of recharge than those estimated by global hydrological models (e.g. Döll and Fiedler 2008), particularly where rainfall is less than 1,000 mm. In areas with extensive clay soils (e.g. central Bangladesh), studies have indicated that groundwater recharge is lower than where the soil is more permeable (Goel et al. 1977; Shamsudduha et al. 2011, 2015).

#### Irrigation transport losses

Indirect recharge from irrigation canal leakage can be significant in areas with a high density of irrigation canals, giving annual recharge of up to 400 mm. This recharge is particularly important in the Indus where rainfall decreases significantly downstream, and in the Ganges, particularly during flood events (Fig. 9).

Across the Indus and Ganges canal systems there are more than 80,000 km of distributor canals overlying the alluvial aquifer system: 59,000 km on the Indus and approximately 25,000 km on the Ganges (Quereshi et al. 2008; FAO 2012, FAO Aquastat 2014) which distribute water to tertiary canals that, in turn, convey water to the fields. Detailed studies of conveyance losses in the tertiary canals in the Indus suggest that losses from the canals are in the range of 0.7–1.6 L/s per 100 m, with lined sections of the canals having slightly lower losses than unlined canals (Clyma et al. 1975; Arshad et al. 2009; Raza et al. 2013). However, the losses increase with the age of the lining and experiments have shown that within 10 years the conveyance losses can rise to the same as unlined canals (Raza et al. 2013). Studies in India (WWF 2015) indicate similar losses—up to 50% of irrigation water is lost through the entire canal network with the vast majority of this water becoming groundwater recharge, and a smaller proportion evaporating. The scale of the groundwater recharge provided by irrigation canals is corroborated by the widespread evidence of water table rise and subsequent waterlogging throughout the 20th century (Quereshi et al. 2008; Basharat et al. 2014; MacDonald et al. 2016).

#### Recharge by the major rivers

The Indus, Ganges and Brahmaputra major river systems form important water resources in the Indo-Gangetic region themselves, with large annual surface water discharges. Prior to development of widespread irrigation across the IGB aquifer, recharge through losses from the river system was a major source of recharge to parts of the aquifer, particularly in the lower Indus where rainfall decreases downstream. The influence of groundwater recharge from the Indus River is observed today by the presence of freshwater in a 50-km buffer zone around the major rivers. In some places the influence is wider due to migration of the river channels. Reducing river flows in the Lower Indus has arguably had an impact on the salinity of the groundwater in the Sindh and consequently the ecology of the mangrove swamps (Qureshi et al. 2010). During the monsoon season, groundwater recharge also occurs close to the Ganges River System, where extensive flooding infiltrates shallow horizons of the aquifer. For much of the year, however, the Ganges river system receives water from groundwater as baseflow, rather than providing recharge.

#### Irrigation field losses

Groundwater is also recharged in irrigated areas from application of excess water to the crops, leading to infiltration of water that cannot be taken up by the plants. Across much the IGB deficit irrigation is practiced (Jurriens and Mollinga 1996); however, some proportion of irrigated water is likely to return to the groundwater, particularly where flood irrigation is practiced. Although providing useful recharge, the returning water can have elevated nitrate concentrations and high salinity since the recharging water flushes out salts within the soil and, if sourced from groundwater, will be more mineralized in the first place (e.g. Ó Dochartaigh et al. 2010).

#### Induced recharge

There is growing evidence that increased pumping in areas with shallow water-tables and permeable soils induces groundwater recharge by creating significant vertical head gradients (Shamsudduha et al. 2011). This behaviour has led some to investigate the possibility of deliberately lowering groundwater levels in the dry season to increase infiltration during the monsoon to help control flooding and increase the water available for irrigation. These ideas were first published in the 1970s within an idea called the *Ganges Water Machine* (Revelle and Lakshminarayana 1975) and have recently been revisited (Khan et al. 2014).

## Hydrogeological typologies

Rather than being a single homogenous aquifer unit, the aquifer properties, hydrochemistry and groundwater recharge of the IGB aquifer vary significantly. Much of the large-scale spatial variation at the basin scale is systematically related to the large-scale changes in sedimentology of the alluvial aquifer, climate, and irrigation practices. The new basin-wide maps illustrate the variability in key aquifer properties within the aquifer system. The systematic changes in the aquifer system which result from these properties are highlighted and described by seven major typologies. Four minor typologies at the margin of the basin accompany the over-arching major typologies.

**Fig. 10:**
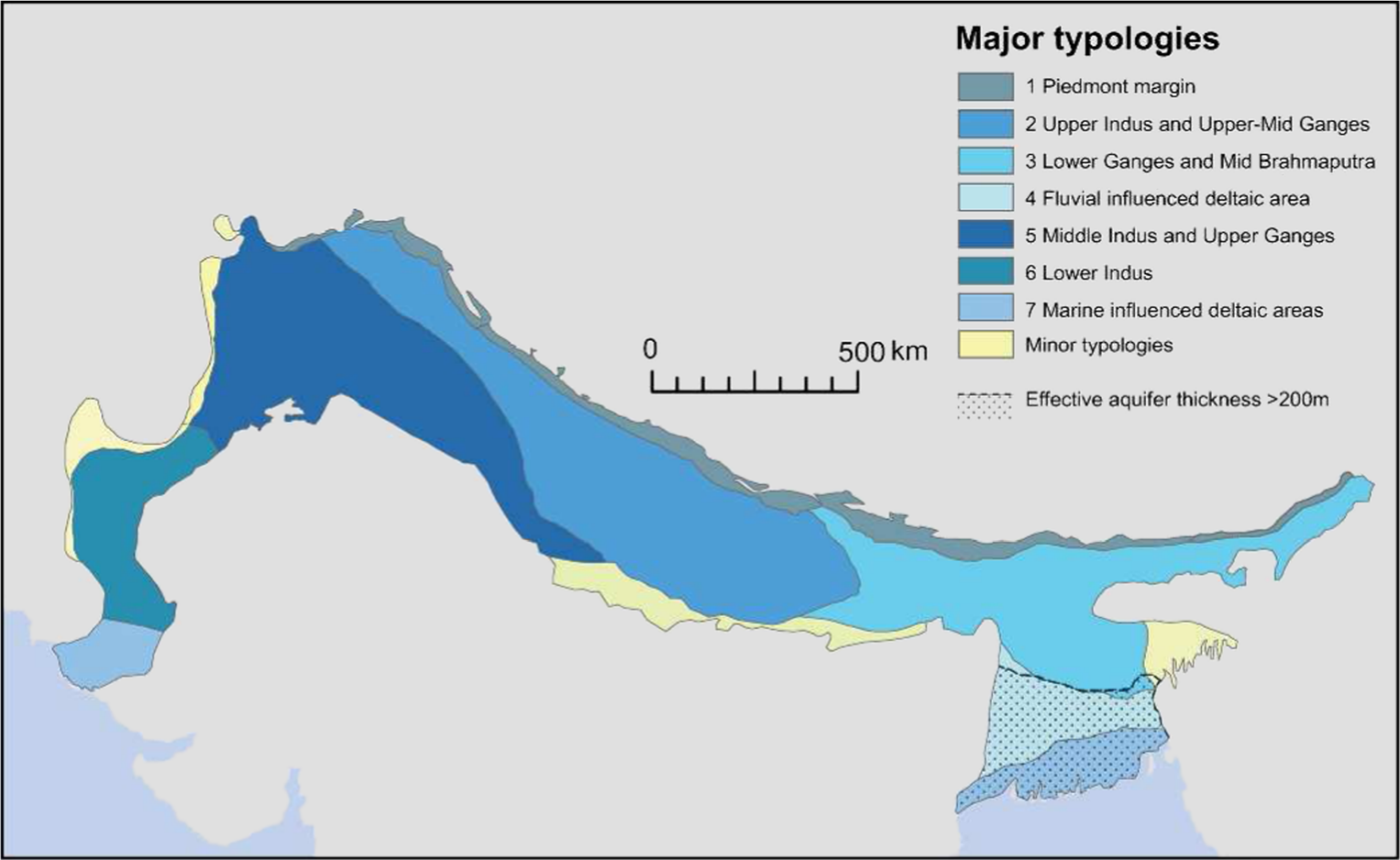
The main groundwater typologies of the Indo-Gangetic basin. (Coastline outline provided by ESRI)

The seven major typologies of the IGB alluvial aquifer system are: (1) the piedmont margin; (2) the Upper Indus and Upper-Mid Ganges; (3) the Lower Ganges and Mid Brahmaputra; (4) the fluvially influenced deltaic area of the Bengal basin; (5) the Middle Indus and Upper Ganges; (6) the Lower Indus; and (7) the Marine influenced deltaic areas. The distribution of the typologies is presented in Fig. 10, and the major characteristics of each of the seven typologies are summarized in Table 1. Each of the typologies is described and illustrated in the proceeding section.

### Typology 1: the Piedmont

The Piedmont is split into three sub-typologies: the main piedmont margin of the IGB aquifer along the edge of the Himalayas; the lower piedmont plain; and the inner valleys.

#### 1A: main piedmont margin

**Fig. 11:**
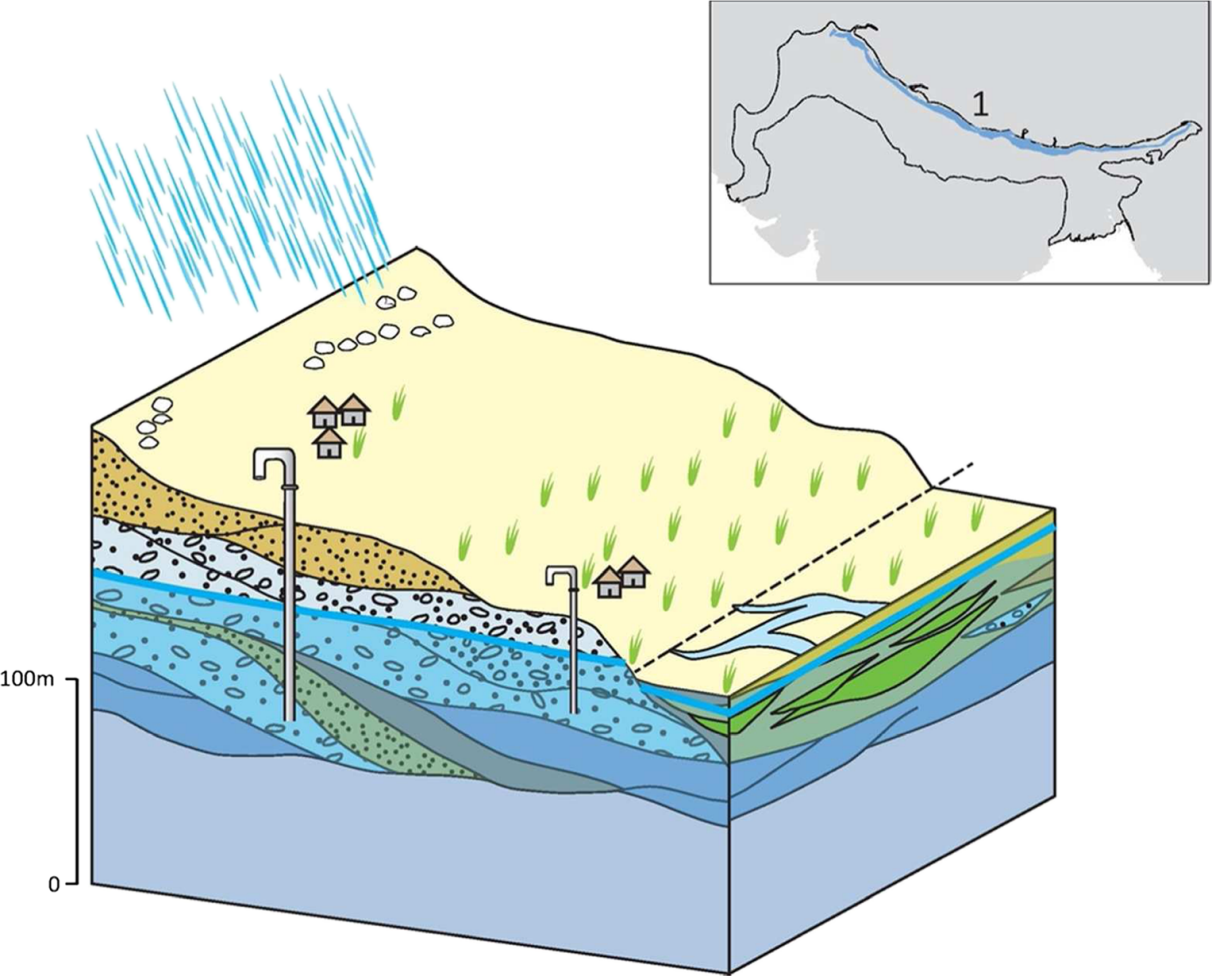
Schematic illustration of the main piedmont margin typology and its distribution. (Coastline outline within inset provided by ESRI)

The main piedmont margin forms a distinct narrow band of very high permeability coarse deposits of variable thickness that extend tens of km along the majority of the northern margin of the IGB aquifer in India and Pakistan at the edge of the Himalaya Middle Hills (Lovelock and Murti 1972; GDC 1994; Fig. 11). The hydraulic conductivity of the aquifer is variable, ranging from 1 to 50 m/d as a result of the poorly sorted sediment, but specific yield is high, typically 20–30% (Lovelock and Murti 1972; Geoconsult 2012). Much of the abstraction occurs from shallow tube wells up to 50 m deep, where yields can be 5–15 L/s; higher yields are common in deeper tubewells (50–80 m deep) where yields of up to 40 L/s are reported (Geoconsult 2012). On valley sides, and in higher elevation intermontane settings, there remains widespread reliance on diffuse hillslope springs. Generally, the sediments are often not more than 150 m thick, and span tens of kilometres north to south. In the context of the IGB aquifer, the effective thickness of this typology is, therefore, relatively thin.

There is high rain-fed recharge to the typology, and significant infiltration and subsurface flow through areas of higher permeability, which generate a distinct spring line at the southern edge of the typology at the junction to the lower terrace of piedmont deposits (typology 2; Narula and Gosain 2013). Groundwater quality is generally good, with little saline groundwater at any depth in the aquifer typology, but elevated As concentrations (>10 μg/L) exist within some parts of the typology (Shrestha et al. 2004; Gurung et al. 2005; Pokhre et al. 2009). The controls of this arsenic contamination are thought to relate to the sediment provenance and the sediment size (relating to the depositional processes) as well as redox conditions. Depth to groundwater is very variable and often relatively deep away from rivers. Typically, the depth to groundwater is 10–30 m but occasionally over 50 m deep in some inter-montane valley systems and away from major rivers.

#### 1B: lower piedmont

**Fig. 12:**
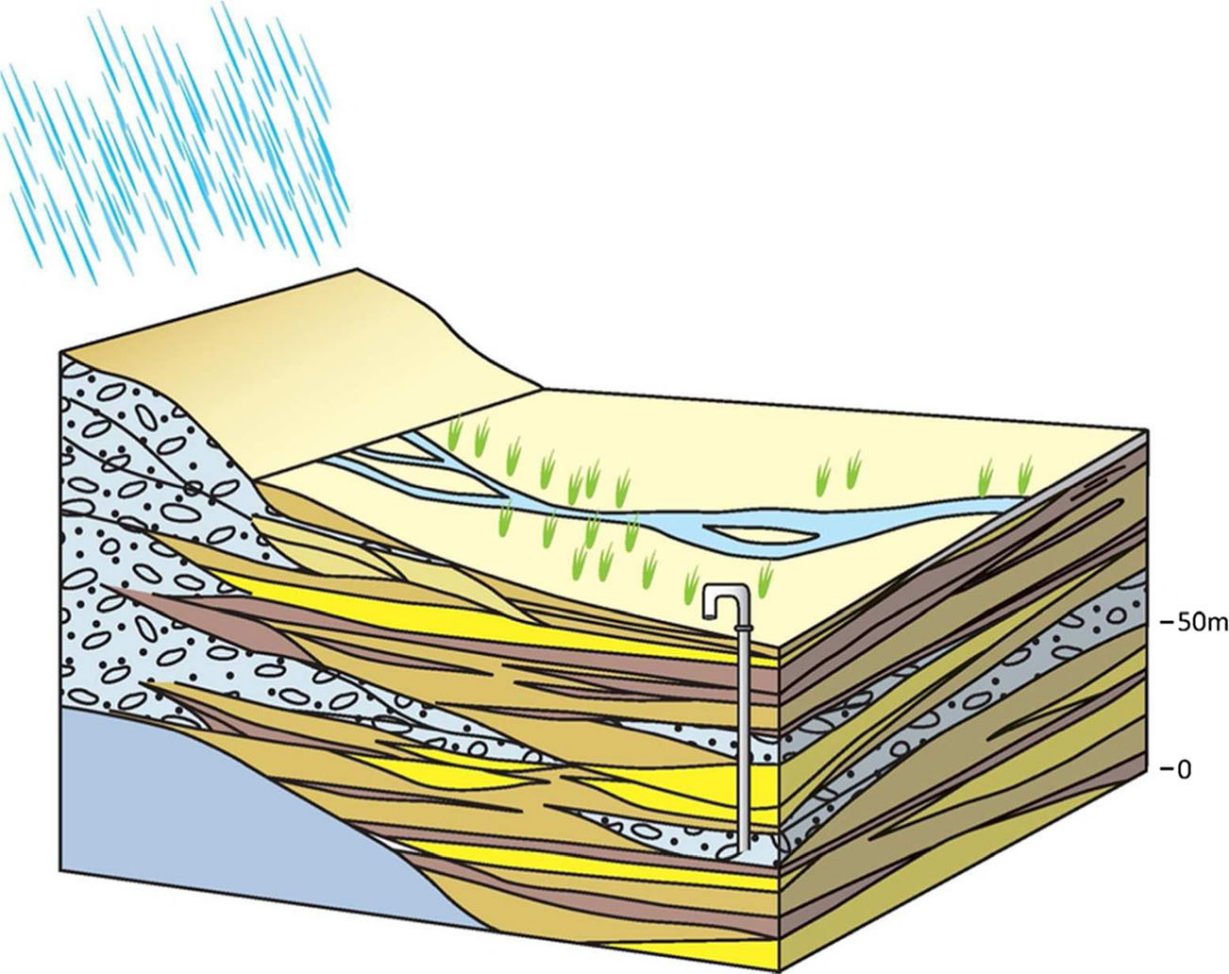
Schematic of the lower piedmont typology. (Coastline outline within inset provided by ESRI)

This lower piedmont alluvium (referred to as the ‘Terai’ in Nepal) is situated closer to the IGB plain and has distinct differences in hydrogeological properties from the upper piedmont typology (Fig. 12). The typology is characterized by a significantly higher proportion of finer alluvium sediments, with up to 20–30% silts and clays, as a result of the slightly lower energy of the fluvial systems which have deposited the alluvium stratigraphy, and the larger distances between active river channels at any one time on the lower piedmont terrace. The proportion of sands and gravels, to silts and fines, at any one place is variable and entirely dependent on the location of past and present channel deposits in the stratigraphy. Horizons of silts and clays relating to the inter-channel depositional processes are present throughout, and laterally continuous for hundreds of metres. The permeability of the IGB aquifer in this sub-typology is therefore typically lower than in the upper main piedmont typology. Hydraulic conductivity is typically 5–30 m/d and shallow tubewells widely provide yields of 5–15 L/s, whilst deeper tubewells 50–80 m deep typically yield 10–40 L/s (Geoconsult 2012). Depth to groundwater in the typology is much shallower than in the coarser upper inter-montane typology, generally 5 m below ground level (m bgl). Artesian groundwater is also common in deeper aquifer horizons as a result of silt and clay horizons producing semi-confined horizons, and mapped to occur across approximately 20% of typology.

#### 1C: inner valleys

**Fig. 13:**
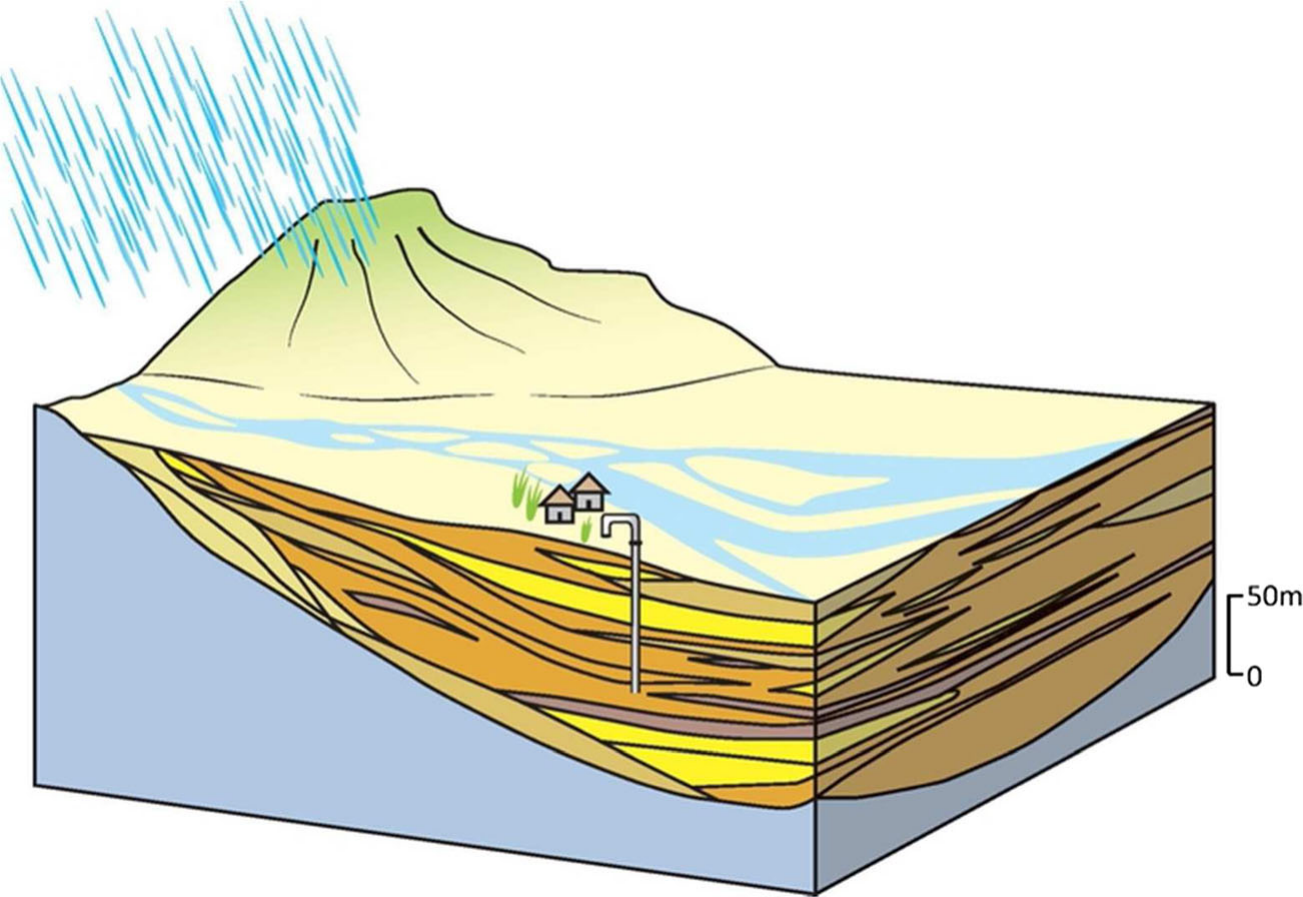
Schematic of the enclosed inter-montane typology environment. (Coastline outline within inset provided by ESRI)

Enclosed valley settings in the lower piedmont, called ‘inner valleys’, are present in a few areas (e.g. in Bhairawa and Nepalganj, Nepal). The groundwater resource is of significantly lower potential in these small disconnected valleys, as a result of the limited spatial extent of the alluvial aquifer and the absence of a significant upper piedmont terrace (Fig. 13). The permeability of the alluvium is also significantly less as a result of the lower proportion of active river channels in the valleys, and the higher predominance of fine sands and silts. Shallow tubewells yields are typically only 1–10 L/s (Geoconsult 2012).

### Typology 2: Upper Indus and Upper-Mid Ganges IGB plain

**Fig. 14:**
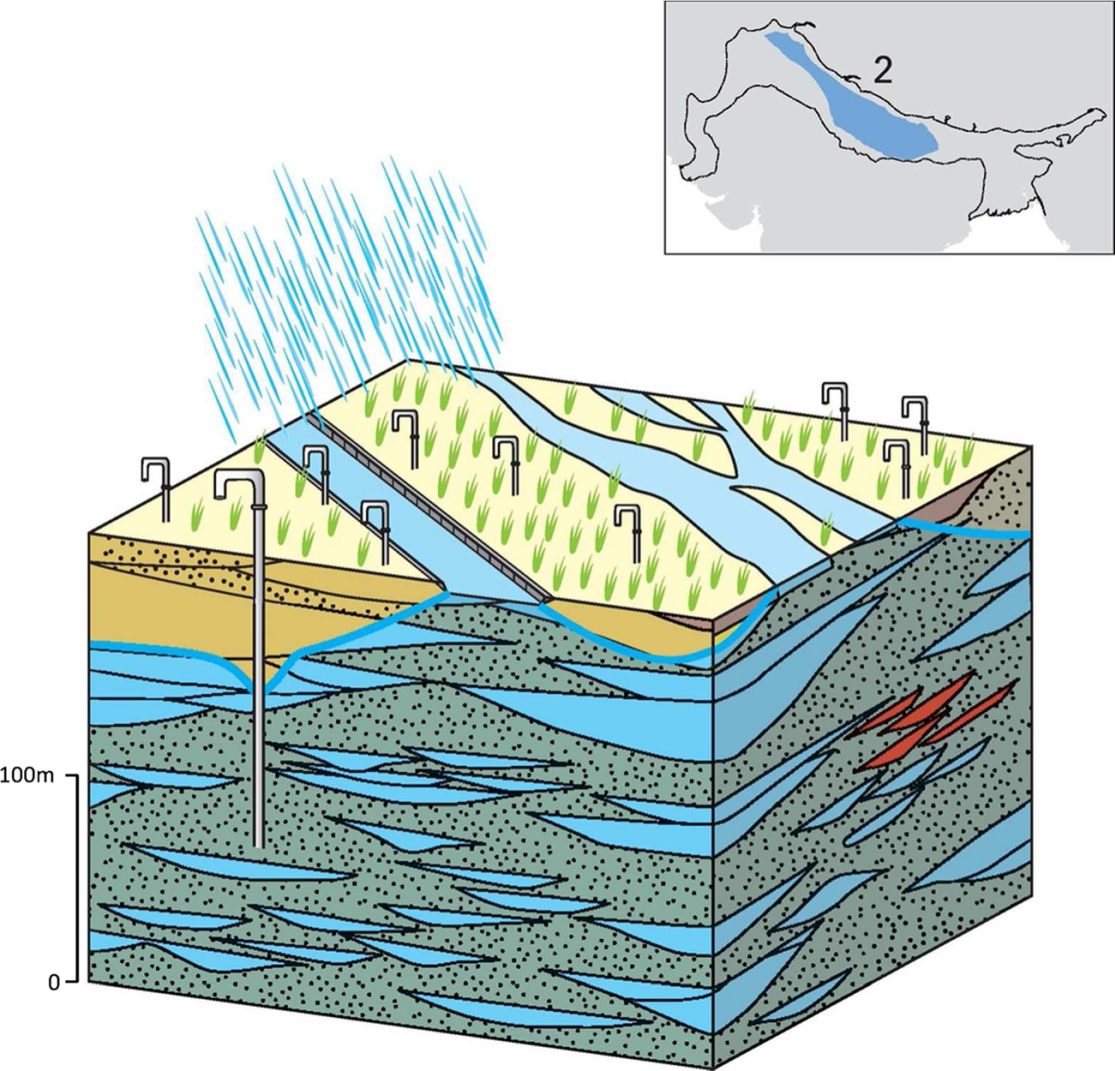
Schematic of the Upper Indus and Upper-Mid Ganges IGB aquifer typology, with* red shading* depicting saline groundwater. (Coastline outline within inset provided by ESRI)

This typology describes a major part of the IGB aquifer system, extending across the Upper Indus and Upper-Mid Ganges basin areas. The typology comprises an extensive highly permeable aquifer with good quality groundwater that runs from the Upper Indus through to the Upper Ganges and Mid Ganges Basin (Sinha et al. 2005b, c; Fig. 14). In total, it covers a spatial area of more than 390,000 km^2^ and is over 200 m thick. The typology is highly exploited with many shallow tube wells (<100 m), hand dug wells and a growing number of deeper tube wells (100–150 m; CGWB 2007). The relatively high groundwater recharge in the typology, from rainfall and canal leakage, mitigates some of the very high abstraction pressures in the typology.

The typology is composed almost entirely of older Pleistocene-aged alluvium, consisting of medium-to-coarse-grained oxidized sands with a high hydraulic conductivity: typically 30–50 m/d, but varying from 10–30 m/d (in the finer inter-channel deposits which contain a higher proportion of fine sands and silts) to 50–70 m/d (in sand dominated channel deposits). The typology has a high specific yield of 10–25% (Chaudhri 1966; Bennett et al. 1969; Niwas and Singhal 1985; Shukla et al. 2001; CGWB 2007).

At a local scale, aquifer properties can vary significantly over short (100s of m to km’s) distances and thicknesses. Low-permeability layers stratify the aquifer, but are rarely continuous over more than a few kilometres (Singh et al. 1999; Samadder et al. 2011; Srivastava et al. 2003), and the anisotropy ratio (i.e. the ratio of vertical to horizontal hydraulic conductivity, Kv/Kh) of the typology varies from approximately <25 in the Upper Indus to 50–100 in the Middle Ganges (Malmberg 1975; Grey et al. 1979; Srivastava et al. 2003). Depth to groundwater is very shallow within the upper alluvium horizons; artesian groundwater conditions are common in the lower aquifer units (more than 50 m below ground surface) with lower-permeability horizons generating leaky, semi-confined aquifer units at depth.

Relatively high recharge to the groundwater resource mitigates some of the high abstraction pressures in the typology. Groundwater recharge occurs through both rainfall and canal seepage (CGWB 2007). Rainfall is high, generally >750 mm, and there are many canals throughout this typology and much of the area is irrigated from both surface water and groundwater (Dhiman 2012).

The groundwater quality of this typology is generally good and it is characterized by substantial renewable groundwater resources. Natural elevated arsenic concentrations can occur in various localities and are usually associated with younger Holocene deposits which have been deposited by modern-day rivers. Groundwater can also be contaminated due to the intensive agriculture and urbanization (Lawrence 1985; CGWB 2007; Saha et al. 2011; Basharat 2012). The presence of saline groundwater is not a major problem but it may occur at shallow levels as a consequence of water logging or in pockets at depths associated with evaporite sequences deposits which developed during earlier periods of lacustrine (lake) environments in the IGB, during periods of drier climates and reduced rates of sediment input and river discharge (Goodbred and Kuehl 2000; Valdiya 2002; Basharat 2012). Elevated fluoride concentrations (>1.5 ppb) place some constraint on groundwater quality in the typology along the southern margin of the upper Ganges basin. Alternating horizons of low and high fluoride concentrations at depth in the aquifer are ascribed to originate from recharge processes in wetter and drier climatic phases, and preferential dissolution of fluoride-bearing minerals from particular alluvium layers.

**Fig. 15:**
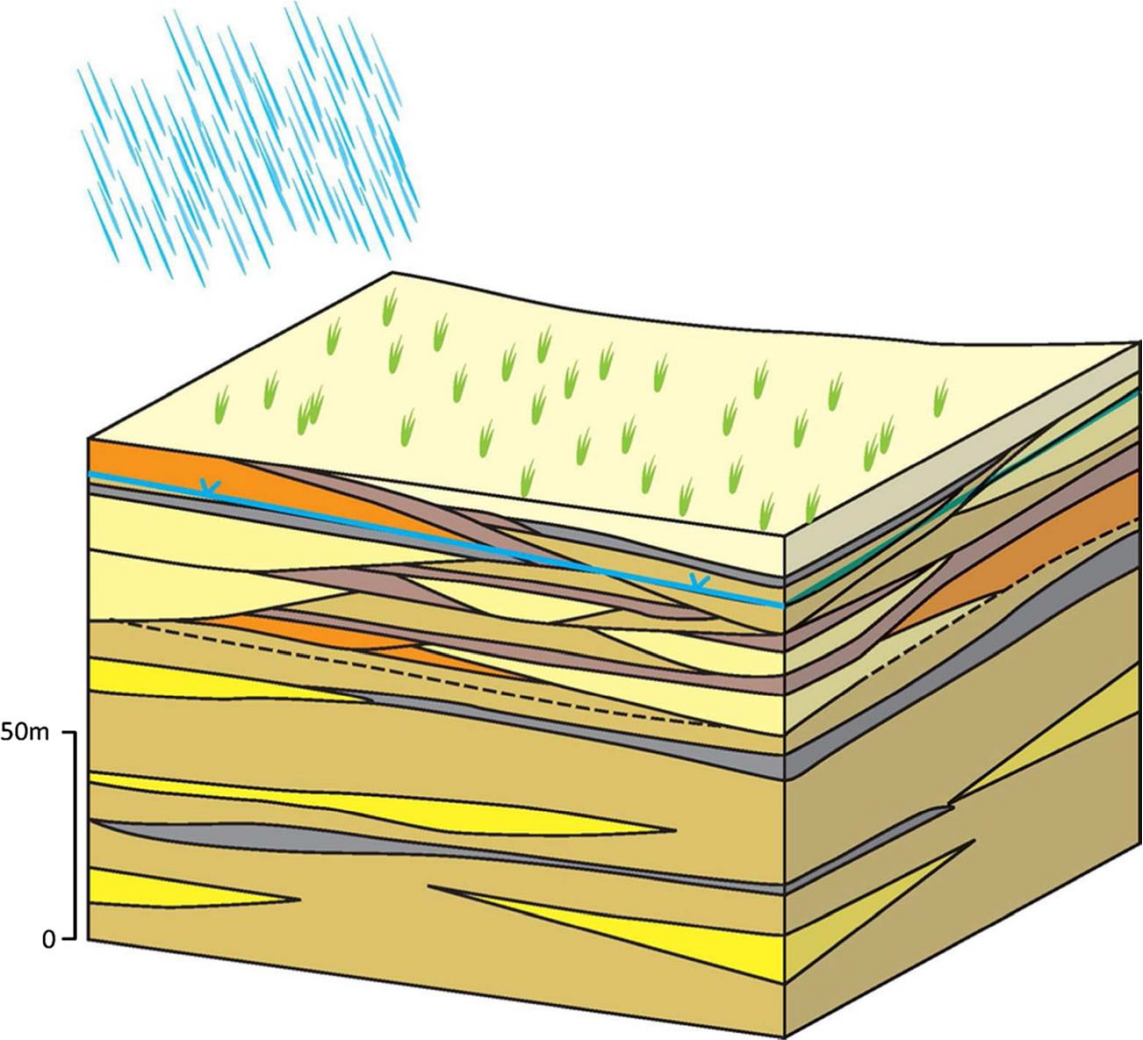
Schematic of the overlying megafan environment within typology 2. (Coastline outline within inset provided by ESRI)

Along the northern margin of the typology, multiple coarse alluvial megafan deposits from the piedmont overlie the Pleistocene typology and form areas of notably higher bulk permeability, greater depth to groundwater (up to 30 m bgl) and greater groundwater potential (25–40 L/s in tubewells 10–60 m deep) than the surrounding typology of the Upper IGB plain (Fig. 15). The largest megafans (the Yamuna-Ganga, Sarda, Gandak and Kosi) are generally over 150 km long, 50–100 km wide, and 50–100 m thick.

### Typology 3: the Lower Ganges and Mid Brahmaputra IGB plain

**Fig. 16:**
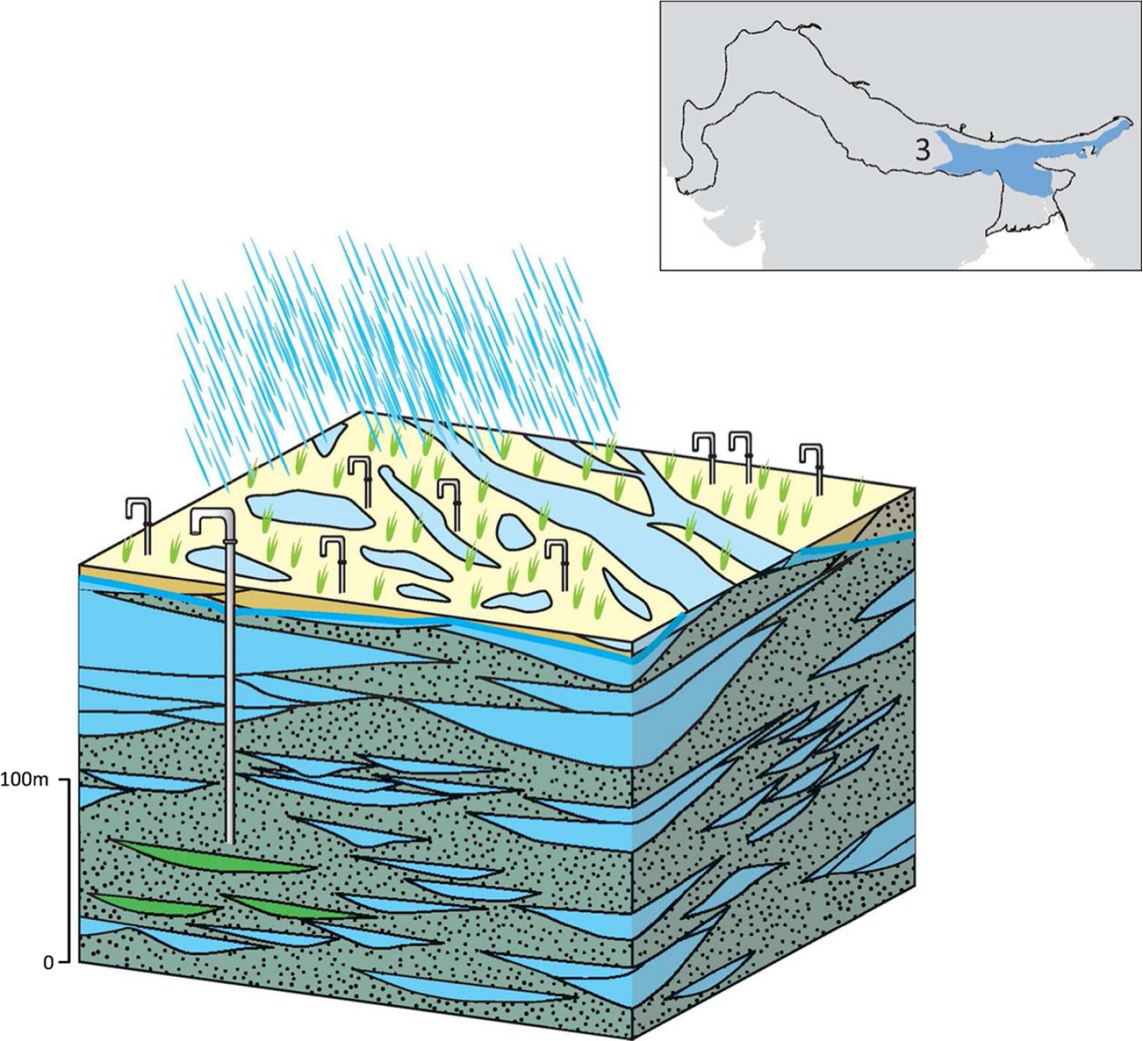
Schematic of the Lower Ganges and Mid Brahmaputra aquifer typology, green shaded areas depicting elevated arsenic concentrations. (Coastline outline within inset provided by ESRI)

This typology forms the effective aquifer unit across a large part of the eastern part of the IGB aquifer, stretching across northeast India and north Bangladesh. The typology has notable differences in groundwater chemistry and recharge to that of typology 2 within the Upper Indus and Ganges IGB plain (Fig. 16).

The typology is formed of highly permeable, predominantly Holocene-aged fine-medium sand-sized alluvium (Singh 1996; Sarma 2005; DPHE 2007; Micheal and Voss 2009b). Hydraulic conductivities of the alluvium sediment within this typology are typically 40–80 m/d, and specific yields are generally in the range 10–20% (Bennett et al. 1969; BGS and DPHE 2001). Since the aquifer runs along the front of the Himalayas, it still receives sediment input along its length and the anisotropy ratio (Kh/Kv) of the aquifer is similar to that of typology 2, and in the range 25–100 (Michael and Voss 2009b). Similarly, low-permeability units are unlikely to be extensive, extending to only several kilometres areal extent (BGS and DPHE 2001).

Groundwater is widely used in the typology, particularly within Bangladesh. Across the typology, abstraction is widely focused to the upper shallow aquifer horizons 0–50 m bgl, as it is across the IGB, and shallow tubewells in the typology typically provide 20–70 L/s. Deeper tubewells, where employed, often provide higher yields, sometimes >100 L/s, and highlight the potential of the multi-layered aquifer.

There are important differences in the groundwater chemistry and recharge in this part of the IGB aquifer, compared to the Upper Indus and Ganges typology (typology 2). Saline groundwater is not as widespread but elevated arsenic concentrations (often >50 μg/L) are common in localized areas throughout the typology (Harvey et al. 2006; Shah 2008; Kumar et al. 2010; Bhattacharya et al. 2011; Fig. 16). The majority of the soluble arsenic occurs in the form of arsenic (As) III. Highest arsenic concentrations occur where there is a continuous clay layer close to the surface of the upper aquifer and typically at depths of less than 30 m bgl.

Potential recharge to groundwater is high but predominantly from rain-fed recharge and seasonal flooding from rivers, in the absence of canal irrigation across much of the typology (CGWB 2010). Annual rainfall is >1,000 mm across the typology; therefore potential recharge is high and there is evidence that actual recharge is primarily limited by the availability of aquifer storage (Shamsudduha et al. 2011) and the permeability of surface geologies (Shamsudduha et al. 2015). Depth to groundwater is very shallow: typically 2–5 m bgl, or even at ground surface in local areas causing long periods of flooding. Lower aquifer units are often leaky and semi-confined with artesian groundwater, as in typology 2.

### Typology 4: the fluvially influenced deltaic area of the Bengal Basin

**Fig. 17:**
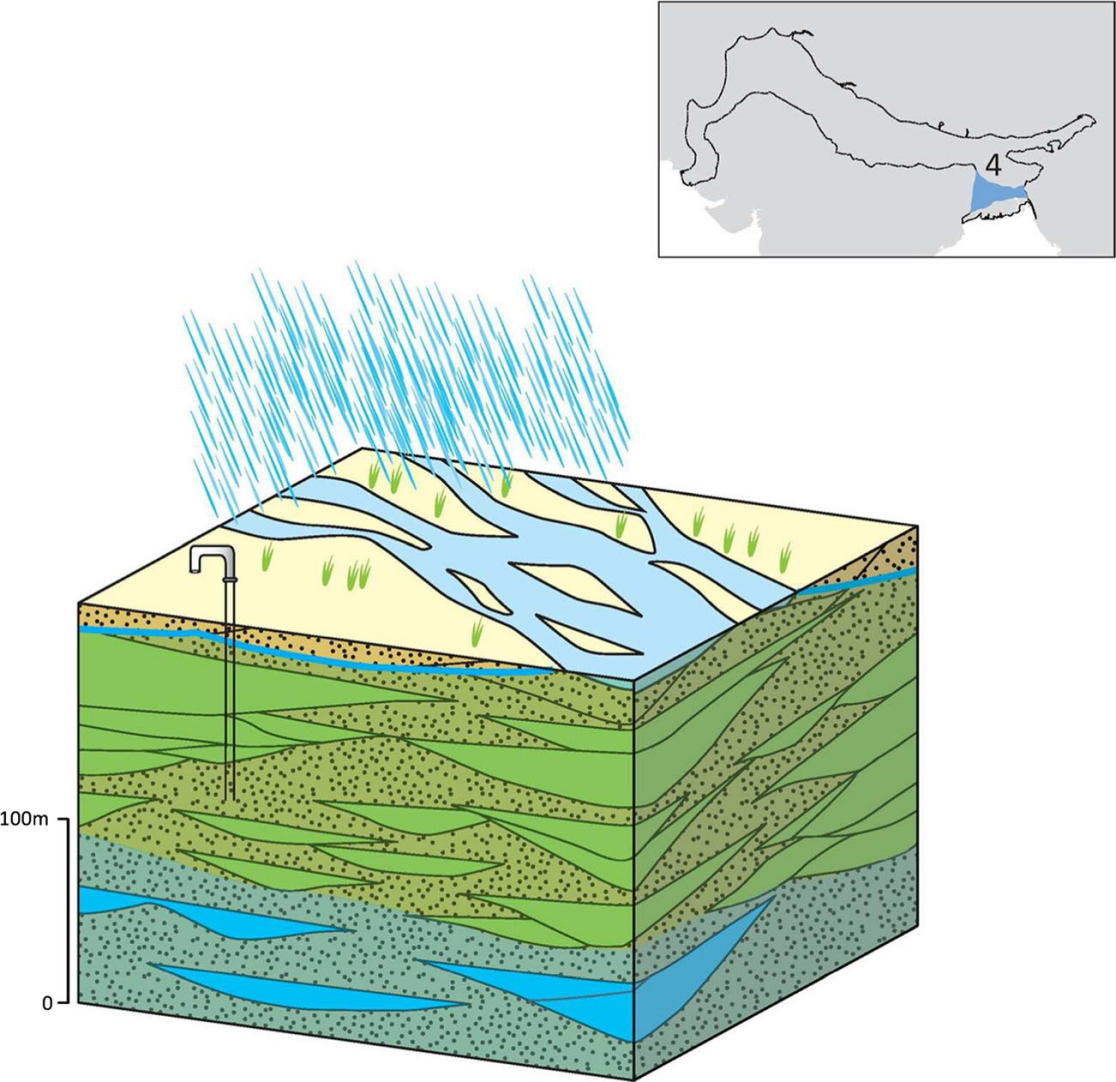
Schematic of the fluvial influenced deltaic area of the Bengal Basin, with* green-shaded areas* depicting elevated arsenic concentrations. (Coastline outline within inset provided by ESRI)

This typology of the IGB aquifer system is distinctive and extends across central Bangladesh. Extremely high concentrations of arsenic in the shallow groundwater resource (generally <100 m deep) mean that there is limited accessible and safe fresh groundwater in the shallower horizons of aquifer despite high rain-fed recharge (Fig. 17). As a result, deeper groundwater (>150 m) forms a strategic resource for water supply and economic development in the region (see section ‘Deeper groundwater in the Bengal Basin’).

The primary aquifer in the typology is formed from Holocene alluvium. Relative to upstream typologies, the Holocene alluvium is composed of a significantly higher proportion of silt and clay (50–70%), and the overall bulk hydraulic conductivity of the aquifer typology is lower, typically 5–35 m/d, and specific yield is <10%. The anisotropy ratio of the Holocene alluvial aquifer is high due to the greater prevalence of low-permeability units in the typology, which are continuous over tens of kilometres (Jones 1985; Allison et al. 2003; DPHE 2007; Michael and Voss 2009b). Mean horizontal permeability is modelled to be 10,000 greater than the vertical permeability (Michael and Voss 2009a, b). Yields from shallow tubewells 0–50 m deep are typically 10–30 L/s. Due to the arsenic contamination within shallow groundwater (0–100 m bgl), abstraction from deeper boreholes (80–200 m deep), which penetrate the underlying Pleistocene alluvium, is increasingly being used for drinking supply. Yields of these deeper boreholes are often >50 L/s and up to 200 L/s indicating the significant potential of the Pleistocene alluvium which contains a higher proportion of coarse sands than the overlying Holocene sediments.

Elevated arsenic concentration in shallow groundwater is widespread and very high concentrations (>200 μg/L) are common (BGS and DPHE 2001; Acharyya 2005; Harvey et al. 2006; Mukherjee et al. 2011). At depths of >150 mbgl, groundwater generally has lower arsenic concentrations. The limited vertical permeability of this highly anisotropic typology is thought to greatly impede vertical movement of the shallow, arsenic-contaminated groundwater. Greater research is nonetheless required to better understand the hydraulic connection between the shallow and deep groundwater and assess whether increased groundwater abstraction from the deep groundwater is able to draw down arsenic in shallow groundwater (Ravenscroft 2007; Michael and Voss 2009a; Burgess et al. 2010). Saline groundwater is also present in the typology extensively throughout the lower Ganges and within shallow groundwater along the margins of the Ganges, Meghna and Madhumati rivers, which suffer seasonal incursion of saline water from the coast.

Potential rain-fed recharge is high as annual rainfall is greater than 2,000 mm across the typology; actual recharge is limited by available aquifer storage and also by the presence of low-permeability soils in some places (CGWB 2007; Shamsudduha et al. 2011, 2015). Deeper groundwater, which forms a strategic resource in this typology due to As-contaminated shallow groundwater, receives little modern groundwater recharge (BGS and DPHE 2001; Shah 2008; Hoque and Burgess 2009; Fendorf et al. 2010; Burgess et al. 2010).

### Typology 5: the Middle Indus and Upper Ganges IGB plain

**Fig. 18:**
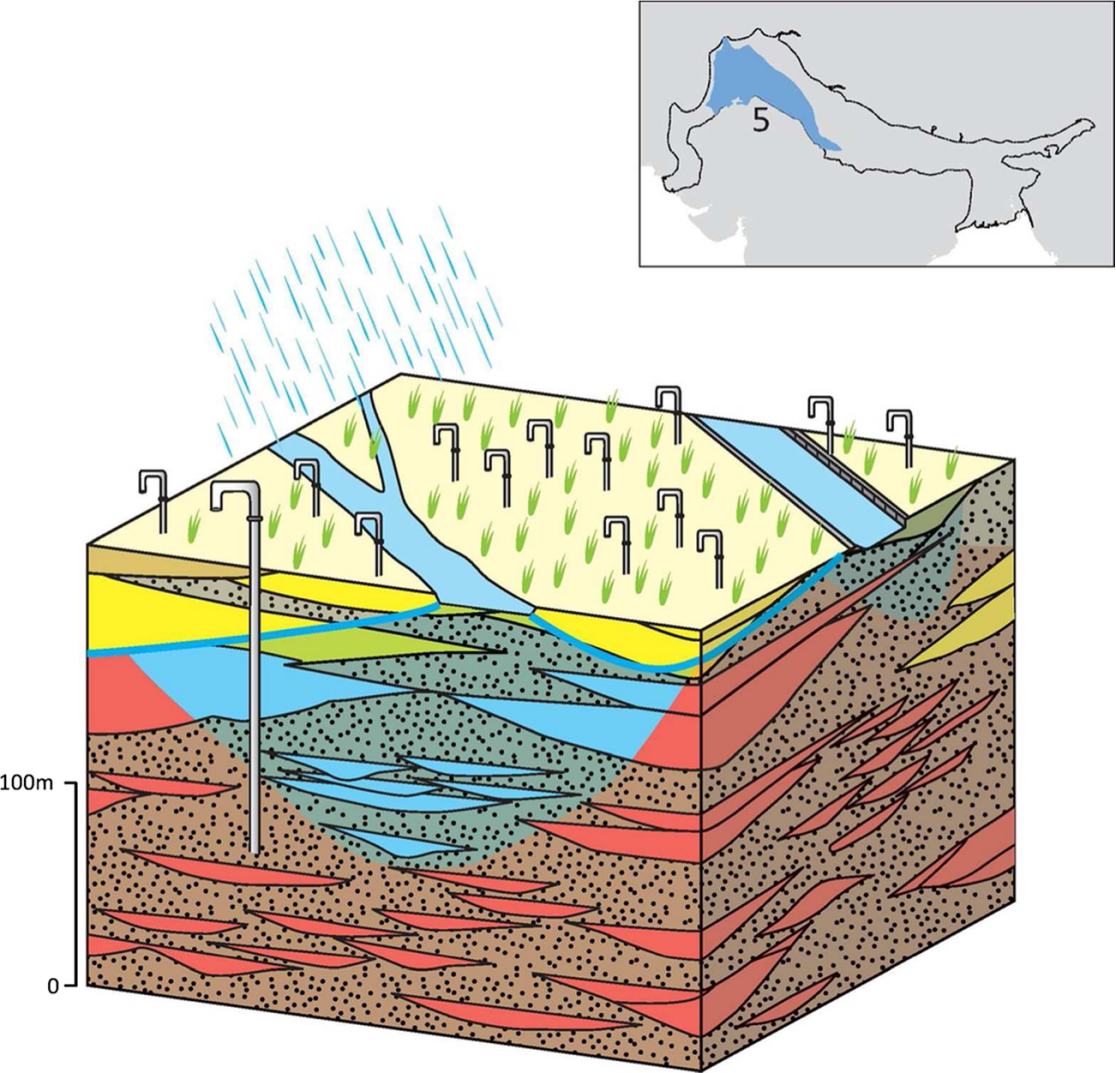
Schematic of the Middle Indus and Upper Ganges aquifer typology, with* red shading* depicting areas of saline groundwater. (Coastline outline within inset provided by ESRI)

The Middle Indus and Upper Ganges typology comprises a highly permeable aquifer stretching across the drier area of the middle Indus and Upper Ganges basins (Fig. 18). The hydraulic conductivity of the aquifer is generally high, often 30–50 m/d and locally up to 50–60 m/d with a high specific yield (10–20%), and with a regional anisotropy ratio of 25–100, though this is much lower in the recent deposits next to modern river channels (Bennett et al. 1969; Sir Mott MacDonald and Partners Ltd. 1982). In contrast to typology 2 within the Upper Indus and the Upper-Middle Ganges, saline groundwater is pervasive in this typology, remote from rivers, and limits fresh groundwater storage available for domestic and agricultural abstraction (Fig. 18). Recharge to the typology is also lower and predominantly from canals and rivers in contrast to the Upper Indus and Upper-Middle Ganges areas, which are predominantly recharged by rainfall. Across the Middle Indus, rainfall is highly seasonal with often less than 25 wet days within a year and average annual rainfall is less than 500 mm; therefore, although rain-fed recharge can occur, it does not dominate. Historically, the aquifer was recharged from the rivers and thick (>100 m) freshwater lenses occur close to the rivers (Sir Mott MacDonald and Partners Ltd. 1985). At present, the aquifer is recharged both from the rivers and the extensive canal network (Basharat 2012; Fig. 18). River flow has diminished due to the high volume diverted to the canal network.

The typology is characterized by pervasive saline groundwater, which restricts groundwater use for abstraction and agriculture. In general, groundwater salinity is < 1,000 mg/L close to the rivers, and >2,500 mg/L away from the influence of the rivers (ISWASRI 2005). Recharge from seepage from the canals can lead to a partial flushing of the shallow groundwater but also to waterlogging and increased salinization in some areas. A complex array of freshwater lenses exists in the upper aquifer, which range from a few metres to tens of metres thick (although generally <30 m) and are laterally extensive over tens to hundreds of metres (Basharat 2012). Elevated natural fluoride and arsenic concentrations as well as nitrate from agricultural practices are common (Gupta et al. 2005). Evaporitic deposits are common within the alluvial stratigraphy and can produce saline groundwater. Groundwater is still extensively used in the typology but subject to greater quality constraints.

### Typology 6: the Lower Indus

**Fig. 19:**
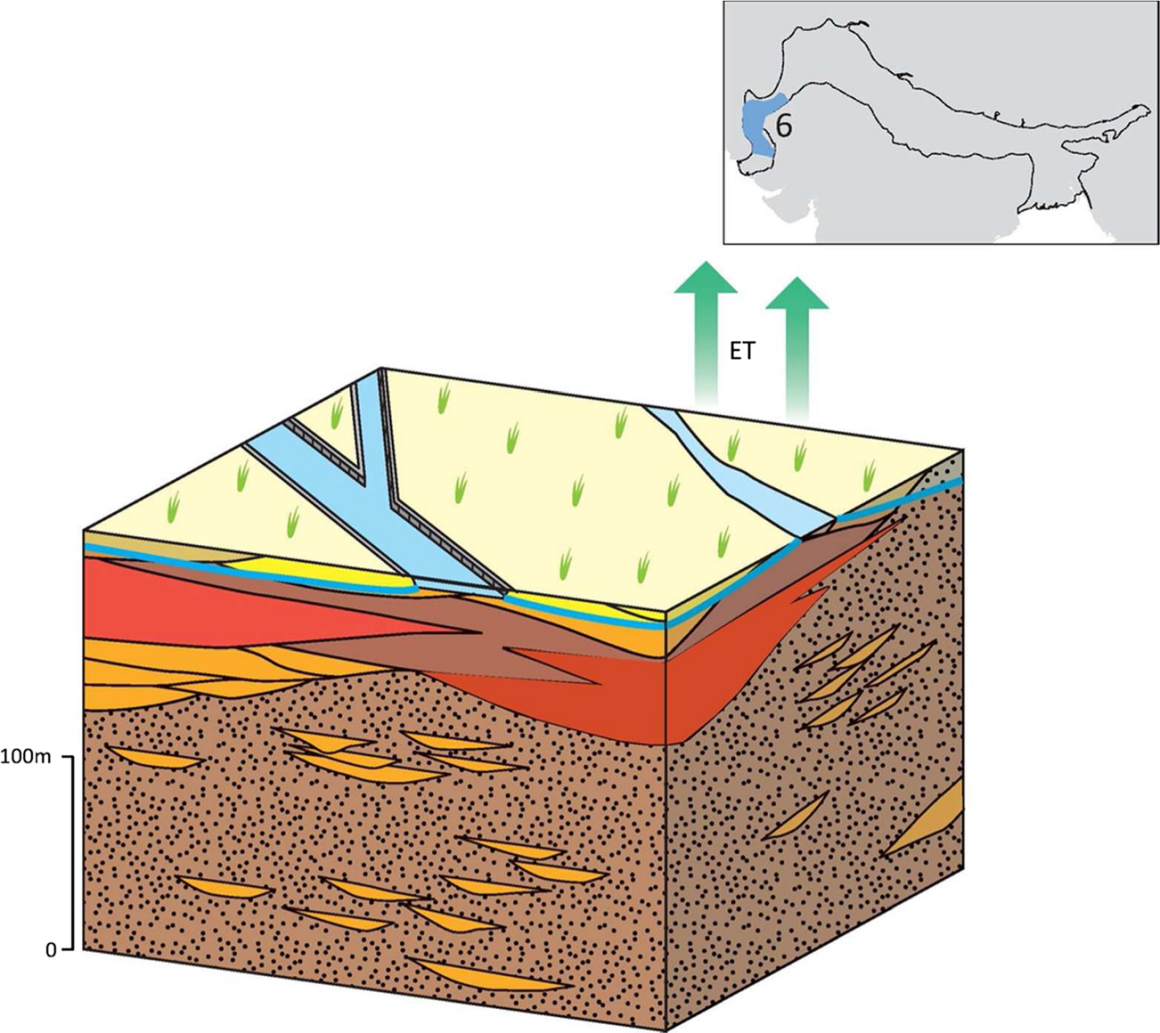
Schematic of the Lower Indus IGB aquifer typology, with* red shading* representing areas of saline groundwater. (Coastline outline within inset provided by ESRI)

The IGB aquifer within the Lower Indus basin in Pakistan is dominated by the presence of pervasive saline groundwater (IWASRI 2005; Fig. 19). Salinity is especially widespread at depth, and the best probability of finding good quality groundwater is at shallower depths adjacent to existing rivers. The high proportion of fine sands and laterally extensive silt layers (Sir Mott MacDonald and Partners Ltd. 1985; Schroder 1993) in the alluvium stratigraphy within this more distal part of the IGB plain, also means the typology is characterized by lower regional vertical permeability and higher anisotropy ratio (Kh:Kv in the region of 100–500) compared to the Upper and Middle Indus-Ganges aquifer typologies (typologies 2 and 5). The hydraulic conductivity of the typology is generally in the range 1–20 m/d, and specific yield ranges from 5 to 15% (Bennett et al. 1969; Sir Mott MacDonald and Partners Ltd. 1990).

In contrast to the lower Ganges (typology 4), which receives significant rain-fed recharge, average annual rainfall in the lower Indus typology is low, <250 mm, and evapotranspiration is high (IWASRI 2005). Groundwater recharge from rainfall is, therefore, negligible. Historically, a larger recharge volume came from the Indus River. This process led to thick lenses >50 km wide of freshwater around modern and paleo river channels (Basharat et al. 2014); however, river flow in the Lower Indus has significantly reduced over the last 40 years due to irrigation and surface-water diversions; recharge from the river now occurs to a more limited extent. Recharge to groundwater in the typology derives predominantly from the canal network and leads to extensive water logging (Sir Mott MacDonald and Partners Ltd. 1994). This indirect recharge has led to the development of thin freshwater lenses in some locations as well as increased phreatic salinization where the water table is very shallow. Groundwater salinity is mostly > 2,500 mg/L; however, next to the Indus, and in localized areas, freshwater lenses can exist and groundwater salinities can be <1,000 mg/L (Sir Mott MacDonald and Partners Ltd. 1985; IWASRI 2005). Evaporite sequences which are common throughout the alluvial aquifer, are a secondary source of saline groundwater locally at depth.

Groundwater is used much less extensively in this part of the basin, and the typology is characterized by an area of groundwater-level rise in the IGB aquifer, in strong contrast to other parts of the basin, where abstraction, groundwater storage potential and recharge are higher.

### Typology 7: marine-influenced deltaic areas

**Fig. 20:**
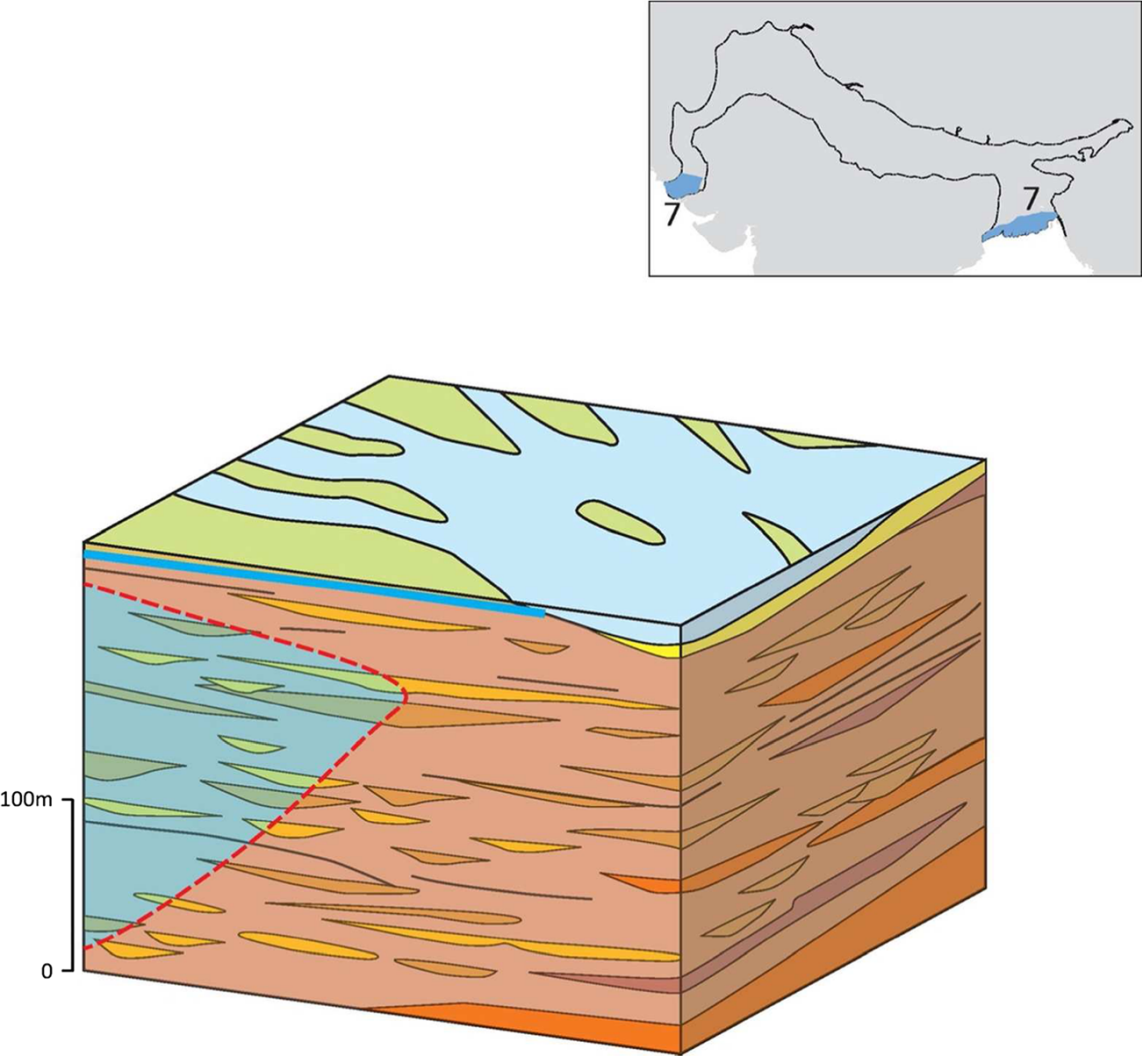
Schematic of typology 7: the marine influence deltaic areas of the IGB aquifer, with the* red-dashed line* and* shading *representing the extent of saline groundwater in the typology. (Coastline outline within inset provided by ESRI)

Both coastal margins of the IGB aquifer represent areas of relatively low fresh groundwater potential in the IGB aquifer system (Fig. 20). Salinity greatly limits the fresh groundwater potential of the Holocene alluvial aquifer within this typology along both the Bangladesh and Pakistan coastal margins. The alluvial aquifer in both coastal margins has the lowest hydraulic conductivity (<10 m/d) and specific yield (<5%) as well as the highest anisotropy ratio (20,000) in the IGB aquifer due to the highly stratified nature of the silt and clay sediments, which were deposited within deltaic or marine-influenced settings. Fluvial channel deposits composed of fine sands are much less prevalent and spatially clustered so that individual channels are typically isolated, rather than connected to adjacent channel units. Whilst groundwater can still flow between the channel units through the interbedded sediments, anisotropy is much greater. Overall, the effective aquifer is composed of approximately 70–80% muds and silts, with only 10–20% sands. Depth to groundwater is shallow, typically <3 m bgl. Yields of shallow tubewells, where they are present, remain around 10–30 L/s. Groundwater is not, however, used extensively in the typology, due to high salinity within the Holocene aquifer. The underlying Pleistocene alluvial aquifer horizons within the typology are typically composed of a higher proportion of coarse sands and can offer greater fresh-groundwater potential, but traditionally have not been extensively exploited.

### Minor typologies

Four minor typologies describe important aquifer variations along the margin the IGB plain aquifer. These include: the deeper groundwater on the Bengal Basin, the southern marginal alluvium in the Ganges basin, the western Indus basin piedmont, and the Sylhet trough in Bangladesh.

#### Deeper groundwater in the Bengal Basin

Within the Bengal Basin, deeper groundwater (150–350 m mgl) is an important resource. Across the rest of the IGB alluvial aquifer system, groundwater is rarely used below 150 m mgl; hence, the restriction of much of this report to discussing the shallowest 200 m. However, due to the widespread contamination of shallow groundwater with arsenic and the evidence of low arsenic concentrations in groundwater > 150 m (BGS and DPHE 2001) in much of the Bengal Basin, deeper groundwater is routinely exploited (Fig. 17). The likely extent of this deeper groundwater is shown in Fig. 10 (DPHE 2007; Michael and Voss 2008; Burgess et al. 2010). The hydraulic properties of these deeper, older sediments are variable but as they are typically composed of a higher proportion of coarse sands, their permeability is higher than that of the overlying Holocene alluvial aquifer. Yields of deeper boreholes which penetrate down into the Pleistocene alluvium are often >50 L/s and up to 200 L/s, indicating the significant potential of deeper groundwater in the Bengal Basin. The aquifer here has been represented in models with an effective horizontal hydraulic conductivity of 40 m/d (Michael and Voss 2008).

Deep groundwater is not subject to recent recharge (Hoque and Burgess 2012) and therefore effective monitoring of abstraction is required to manage the resource, and to ensure that substantial groundwater abstraction from deep in the aquifer is sustainable for decades or centuries to come (Ravenscroft et al. 2013). Careful monitoring is also required to ensure the quality of the deep groundwater is sustainable in the long term since there is concern that intensive abstraction alters vertical hydraulic gradients within the aquifer and may locally draw down younger, relatively As-rich shallow groundwater.

#### The Southern Marginal Alluvium

**Fig. 21:**
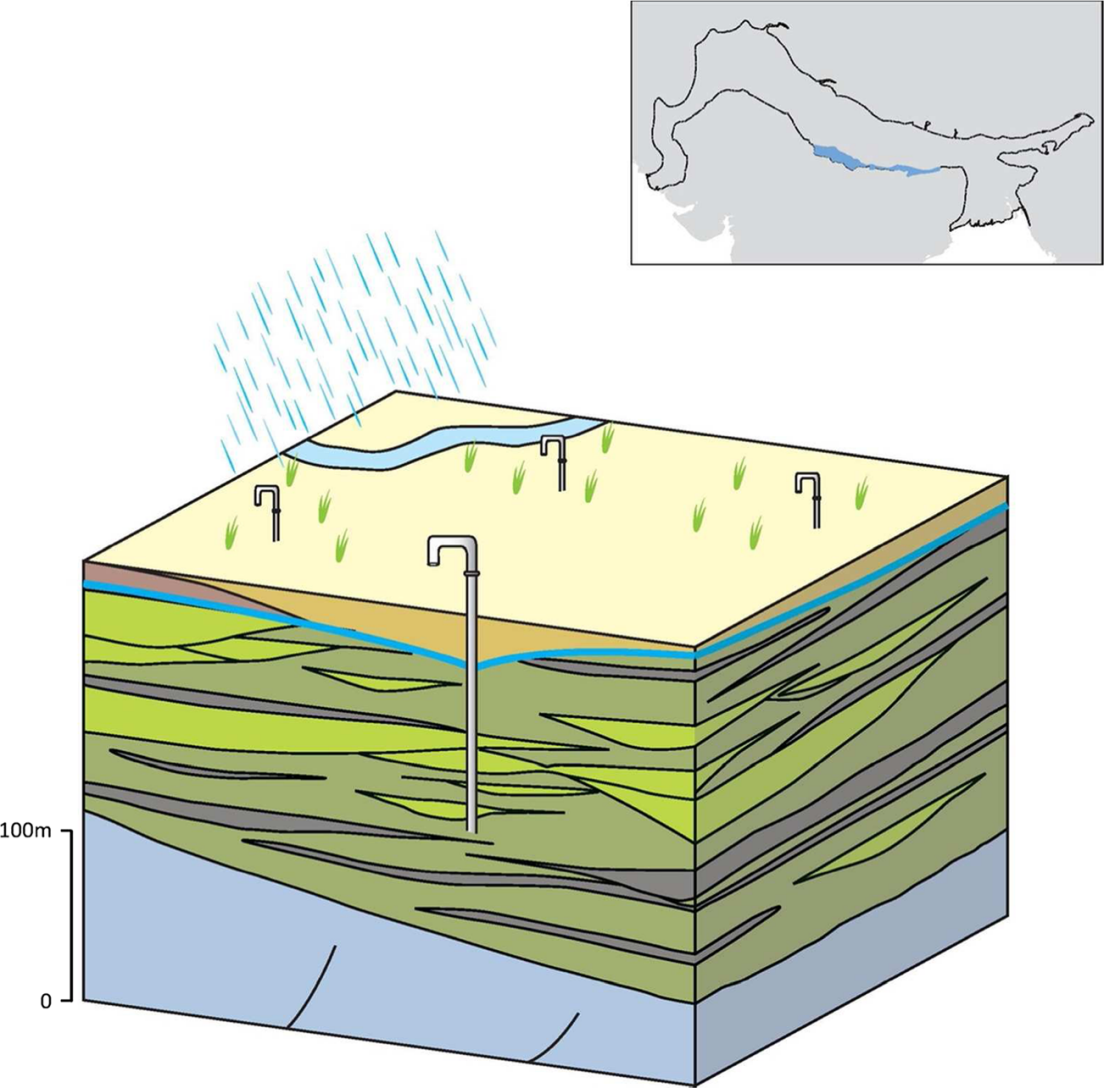
Schematic of the southern margin alluvium typology environment. (Coastline outline within inset provided by ESRI)

The southern marginal alluvial aquifer is located south of the Yamuna River along the southern edge of the upper and central Ganges basin (Fig. 21). It is composed of genetically distinct (smectite-rich) sediment derived from basement and basaltic rocks south of the IGB within the Indian craton (Heroy et al. 2003; Saha et al. 2010; Sinha et al. 2014).The effective thickness of the aquifer within the typology can be significantly less than in central parts of the IGB aquifer north of the Yamuna River (typologies 2 and 3). Along the southern edge of the marginal alluvium plain, the effective aquifer thickness can be less than 200 m and as a result the potential groundwater storage is significantly less (Singh 1996; Heroy et al. 2003; Fig. 21). The permeability and specific yield of the marginal aquifer are, however, similar to that within the adjacent IGB plain typologies (hydraulic conductivity typically 20–60 m/d, and specific yield 10–15%). The natural groundwater quality of the typology is generally good. Shallow groundwater is locally saline (TDS >1,000 mg/L) at the western limit of the typology as a consequence of water logging, or in pockets at depth associated with evaporite sequences deposits under previous climates. Groundwater abstraction is much lower in the typology compared to within the adjacent IGB plain.

#### The Sylhet Basin

**Fig. 22:**
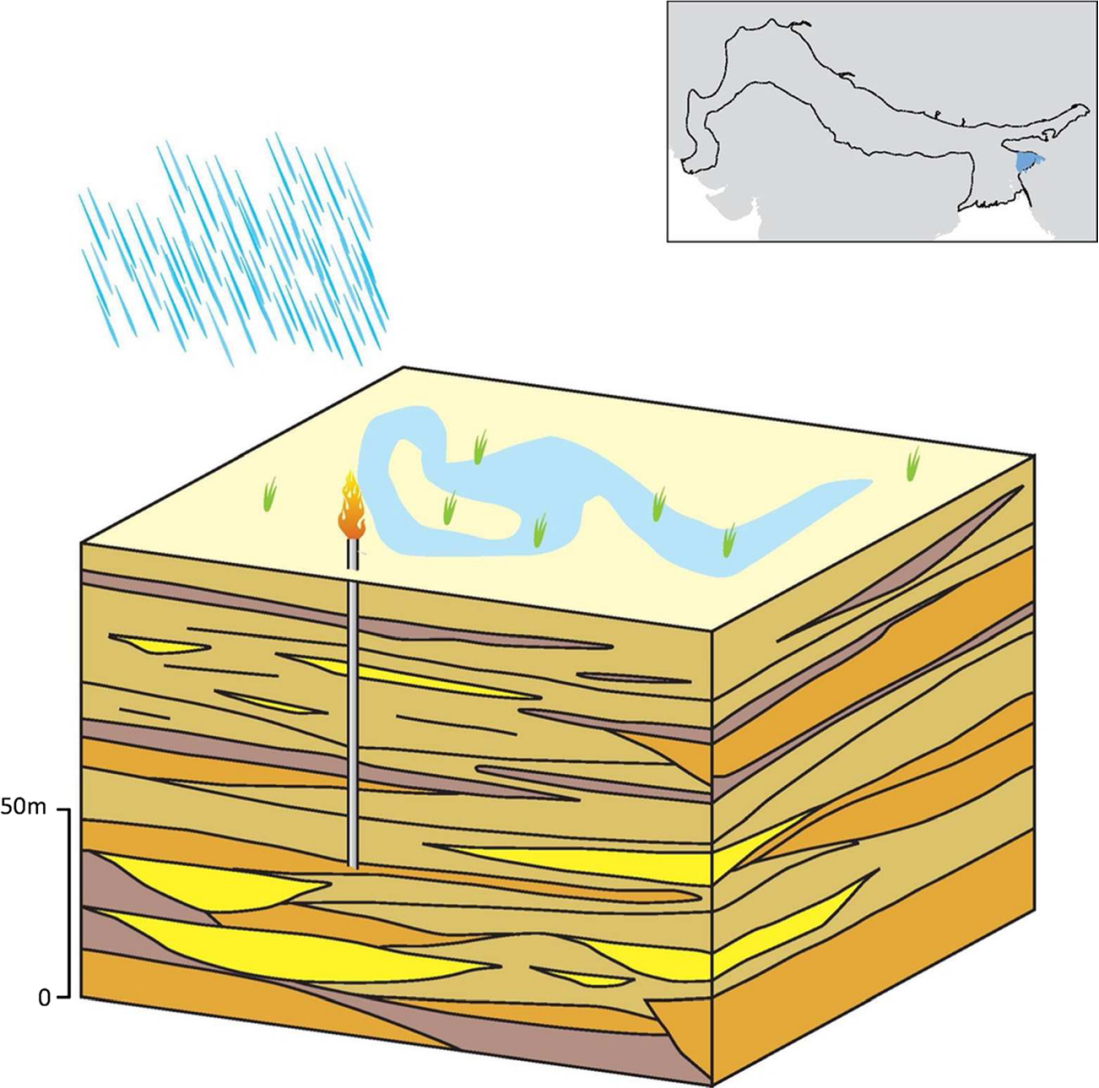
Schematic of the Sylhet Basin typology environment. (Coastline outline within inset provided by ESRI)

The Sylhet Basin forms a discrete typology in the IGB aquifer system which is of significantly lower aquifer hydraulic conductivity <10 m/d and specific yield <5%. The basin is a region of tectonic subsidence, located at the edge of the IGB aquifer in east Bangladesh (Fig. 22). Alluvium within the basin is composed of a high proportion of silts, muds and clays (>60%) deposited in a low energy fluvial and wetland setting (Johnson and Alam 1991; Fig. 22). Higher-permeability channel deposits are typically separated by significant thicknesses (tens of metres) of muds, and individual channel deposits have to be targeted in groundwater development (Johnson and Alam 1991). Depth to groundwater is very shallow (<3 m bgl), with water logging characteristic of the typology. Lower aquifer units are semi-confined or confined and typically have a piezometric head which is above the water-level in the upper aquifer units (BGS and DPHE 2001).

Groundwater abstraction is limited within the typology, as a result of elevated concentrations of methane within groundwater—the Sylhet Basin forming a major gas field in Bangladesh—and also local elevated concentrations of arsenic within shallow groundwater (BGS and DPHE 2001). Abstraction is also limited due to the abundance of surface water from high rainfall.

#### Western Indus Basin piedmont

**Fig. 23:**
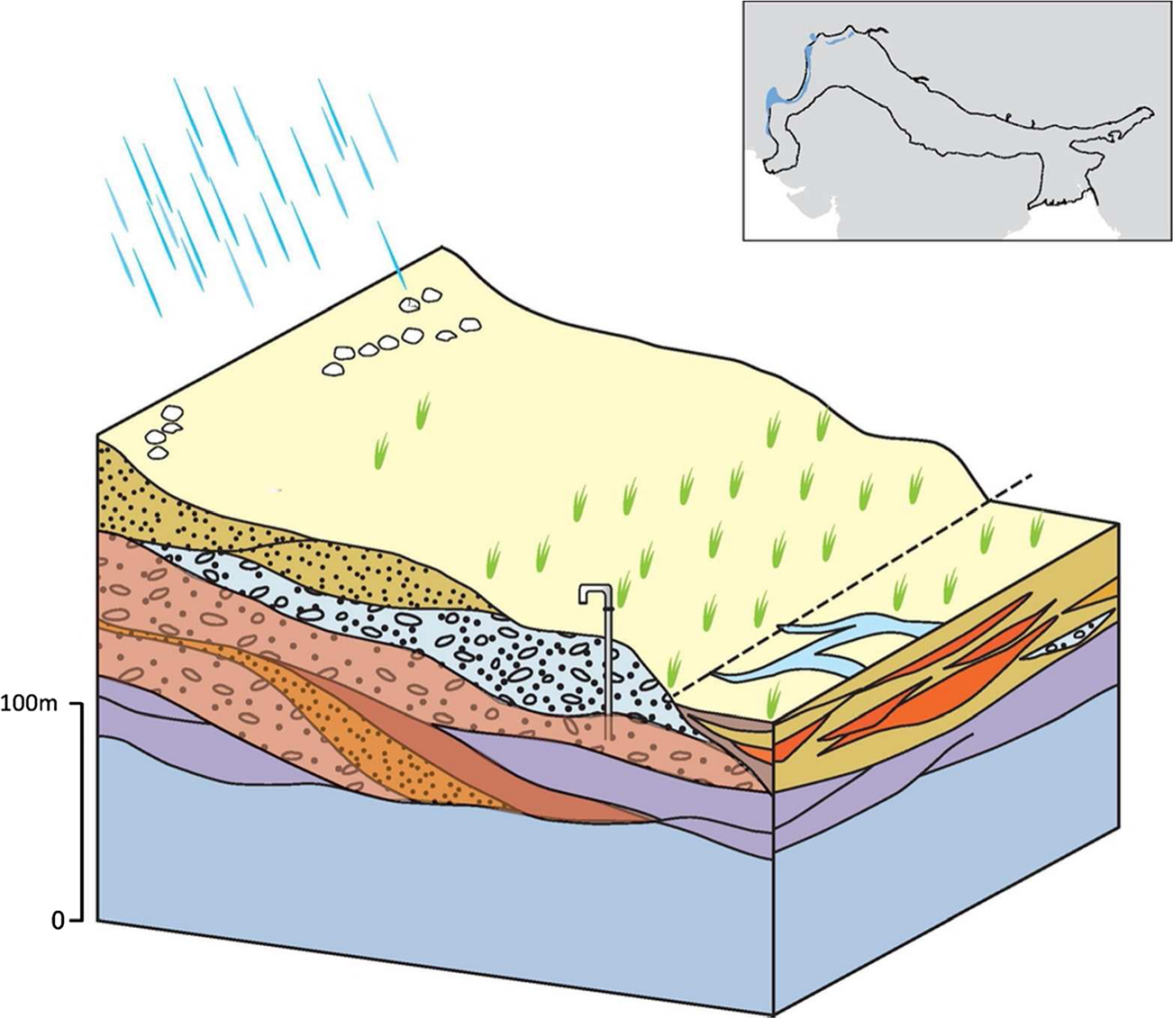
Schematic of the Western Indus piedmont typology environment, with* red shading *representing areas of saline groundwater. (Coastline outline within inset provided by ESRI)

The Western Indus piedmont forms a relatively narrow band of coarse high permeability deposits along the western margin of the Indus basin comparable in many respects to the Himalaya piedmont along the northern margin of the IGB (typology 1; Fig. 23). In contrast to the Himalaya piedmont, however, average annual rainfall is less than 500 mm in the Western Indus and rain-fed recharge is more limited. Groundwater is generally saline but with local exceptions. Localized recharge occurs from rivers and spate irrigation systems along the piedmont. Depth to groundwater is variable but can be locally deep (>50 m).

## Discussion

Each of the seven major typologies represents an area of the alluvial aquifer system with a distinct set of challenges and opportunities for groundwater development. The groundwater typologies clearly illustrate the heterogeneity of the IGB aquifer, with specific dynamics and resilience to future changes in abstraction and climate. From a policy perspective, this variability may necessitate a strengthening of scientific and management capacity at local and regional levels in order to mitigate risks and exploit opportunities.

The IGB aquifer system is among the most important water resources in South Asia. As the typologies indicate, parts of the system are highly resilient and, with appropriate management, could serve as a sustainable source of water for domestic, agricultural and environmental uses despite on-going changes in climate variability, land-use, and groundwater abstraction. At the same time, other areas of the basin are susceptible to over-extraction and/or major water quality issues. As the typologies indicate, aquifer characteristics vary substantially but in ways that generally do not coincide with political or administrative boundaries. In addition to strong local scientific and management capacity, this suggests the importance of state or regional regulatory mechanisms and legal frameworks that enable tailoring of both surface and groundwater use, development, and management, to local conditions within the broader typologies. Policy considerations of this type will play a major role in, for example, the ability to take advantage of major conjunctive use opportunities in the highly resilient typologies 2 and 3, while avoiding the overdraft and water quality risks observed in other typologies.

Overall, by combining several datasets of aquifer storage, permeability, recharge systems and water quality, the typologies provide a new lens through which to conceptualize large-scale aquifer properties, dynamics, and *resilience* of the aquifer system. The typologies can guide appropriate aquifer management strategies. As a starting point, the typologies could be used to help prioritize groundwater monitoring, investigation, and local capacity development as a precursor to policy changes and the eventual development of regionally specific management strategies. Some issues within the IGB aquifer that the typologies help to highlight are discussed further:

### Differences in groundwater storage and permeability: resilience to abstraction

The typologies intentionally do not include information about groundwater abstraction or pollution pressures, but are restricted to the hydrogeological properties which govern its development potential and make it resilient to changes in climate or abstraction (Foster and MacDonald 2015). The high specific yield of the IGB aquifer means that large volumes of groundwater are stored in the aquifer system—potentially, 20 times the annual flow of the river systems (MacDonald et al. 2016); however, the high permeability makes the aquifer vulnerable to over-exploitation. The typologies illustrate where the resilience to depletion is greatest. For example typology 2 (Upper Indus and Upper Mid Ganges) has a high resilience to change due to the high storage and recharge, which has buffered some of the high rates of groundwater abstraction. This resilience suggests opportunities for conjunctive management that would enable substantial storage and recovery of groundwater if the development of surface irrigation systems can be coordinated with areas of high groundwater use or demand. Typology 5, however (Middle Indus and Upper Ganges), is less resilient to change due to the lower long-term groundwater recharge as highlighted by the strong adverse groundwater response to intensive groundwater abstraction (Rodell et al. 2009; MacDonald et al. 2015). Management strategies within this typology will ultimately need to focus much more on management of the risks and consequences associated with high abstraction. Typology 3 (the Lower Ganges and Mid Brahamaputra) has a high resilience to change, and shows few side effects from over abstraction; therefore, there is potential here for further groundwater abstraction and possibly large-scale conjunctive management subject to further investigations.

### Degradation of groundwater quality in the IGB aquifer

Two of the greatest constraints to the useable volume of fresh groundwater in the IGB are the presence of saline water at shallow depths and elevated arsenic concentrations. It is estimated that excessive salinity or arsenic occurs over 60% of the IGB aquifer area and four of the seven typologies are severely affected by these groundwater quality constraints (MacDonald et al. 2016). Specific groundwater management strategies are likely to be essential, based on the local conditions—for example, salinity in the Lower Indus requires a different management strategy to the growing issue of salinity in the Middle Indus and Upper Ganges, or salinity in coastal Bangladesh. Even where groundwater is largely of good quality (e.g. Lower Ganges) and much less vulnerable to salinization or the presence of widespread arsenic contamination (e.g. Mid-Brahmaputra), management measures are required to minimize the growing problem of anthropogenic pollution from industry, cities and agriculture. These challenges again highlight the importance of local scientific and management capacities in order to mitigate groundwater quality risks.

### Recharge management in low rainfall areas

The typologies provide a framework to understand how different management approaches might affect the distribution of recharge in the IGB aquifer and their implications for groundwater quantity and quality. Within areas of the IGB aquifer which receive low annual rainfall—for example, canal leakage dominates groundwater recharge; thus, while policies to line canals and reduce leakage may have a positive impact on water delivery and crop productivity, they often have a negative impact on the groundwater balance through water-logging and subsequent phreatic salinization. Resource management needs to be tailored at the local level to save water through channel linings and focus on areas where return flows are lost to further use, or threaten the quality of drinking water or key environmental flows. The typologies help identify these different impacts of management approaches, and how they relate. Where there is deeper saline water, high abstraction practices can also mobilize older, deeper groundwater into shallow depths, which is evident in the Mid Indus/Upper Ganges typology where high abstraction is being accompanied by increasingly saline water.

### Monitoring programmes

Changes in groundwater quality and groundwater storage within the IGB aquifer system will generally be gradual (multi-annular and decadal), and monitoring is essential for appropriate and timely policy frameworks to be established that enable a managed response. Continued exploration, testing and monitoring of shallow and deeper groundwater and their interaction with surface-water sources across the aquifer system are needed to enable timely management systems to be developed to identify and mitigate further degradation. The typologies, which highlight the current status of the IGB aquifer groundwater resource, as well as the major hydrogeological differences across the system, provide a robust framework to inform different capacity development and monitoring requirements within the different parts of the aquifer, to develop effective management strategies at local and regional levels.

## Conclusion

The hydrogeological typologies systemize groundwater knowledge of the Indo-Gangetic alluvial aquifer system, and assemble the information most relevant to understanding its resilience. The seven major typologies highlight significant spatial changes in recharge, permeability, storage and groundwater chemistry at a transboundary-scale across the IGB aquifer, and provide an alternative conceptualization of this aquifer system which is traditionally mapped as a single homogeneous highly permeable aquifer at a transboundary-scale. The typologies indicate different potential opportunities for management and sustainable development while also highlighting regions where mitigation is important to address depletion and degradation. Given that water quality degradation is likely to pose a greater threat than depletion for many of the typologies, systematic regular groundwater quality monitoring is fundamental to track changes and adopt appropriate management strategies. Strong scientific and management capacity at local levels, along with the legal and policy frameworks necessary to tailor management to local groundwater and surface-water conditions within typologies, will be essential in order to mitigate risks and take advantage of opportunities.
